# Phase I Randomized Safety Study of Twice Daily Dosing of Acidform Vaginal Gel: Candidate Antimicrobial Contraceptive

**DOI:** 10.1371/journal.pone.0046901

**Published:** 2012-10-08

**Authors:** Marla J. Keller, Colleen A. Carpenter, Yungtai Lo, Mark H. Einstein, Congzhou Liu, David N. Fredricks, Betsy C. Herold

**Affiliations:** 1 Department of Medicine, Albert Einstein College of Medicine and Montefiore Medical Center, Bronx, New York, United States of America; 2 Departments of Obstetrics and Gynecology and Women’s Health, Albert Einstein College of Medicine and Montefiore Medical Center, Bronx, New York, United States of America; 3 Department of Pediatrics, Albert Einstein College of Medicine and Montefiore Medical Center, Bronx, New York, United States of America; 4 Departments of Epidemiology and Population Health, Albert Einstein College of Medicine and Montefiore Medical Center, Bronx, New York, United States of America; 5 Departments of Microbiology and Immunology, Albert Einstein College of Medicine and Montefiore Medical Center, Bronx, New York, United States of America; 6 Vaccine and Infectious Disease Division, Fred Hutchinson Cancer Research Center, Seattle, Washington, United States of America; University of Toronto, Canada

## Abstract

**Background:**

Acidform gel, an acid-buffering product that inactivates spermatozoa, may be an effective topical non-hormonal contraceptive. This study was designed to evaluate the safety of vaginal dosing and effects of Acidform on mucosal immune mediators, antimicrobial properties of genital secretions, and vaginal microbiota.

**Methods:**

Thirty-six sexually abstinent U.S. women were randomized to apply Acidform or hydroxyethylcellulose (HEC) placebo gel twice daily for 14 consecutive days. Safety was assessed by symptoms and pelvic examination. The impact of gel on mucosal immunity was assessed by quantifying cytokines, chemokines, antimicrobial proteins and antimicrobial activity of genital secretions collected by cervicovaginal lavage (CVL) at screening, 2 hours after gel application, and on days 7, 14 and 21. Vaginal microbiota was characterized at enrollment and day 14 using species-specific quantitative PCR assays.

**Results:**

The median vaginal and cervical pH was significantly lower 2 hours after application of Acidform and was associated with an increase in the bactericidal activity of CVL against *E. coli*. However, 65% of women who received Acidform had at least one local adverse event compared with 11% who received placebo (p = 0.002). While there was no increase in inflammatory cytokines or chemokines, CVL concentrations of lactoferrin and interleukin-1 receptor antagonist (IL-1ra), an anti-inflammatory protein, were significantly lower following Acidform compared to HEC placebo gel application. There were no significant changes in *Lactobacillus crispatus* or *Lactobacillus jensenii* in either group but there was a decrease in *Gardnerella vaginalis* in the Acidform group (p = 0.08).

**Conclusions:**

Acidform gel may augment mucosal defense as evidenced by an increase in bactericidal activity of genital secretions against *E. coli* and a decrease in *Gardnerella vaginalis* colonization. However, Acidform was associated with more irritation than placebo and lower levels of antimicrobial (lactoferrin) and anti-inflammatory (IL-1ra) proteins. These findings indicate the need for additional safety studies of this candidate non-hormonal contraceptive.

**Trial Registration:**

ClinicalTrials.gov NCT00850837

## Introduction

Several epidemiological studies indicate that systemic hormonal contraception, particularly progesterone-containing injectables, may be associated with an increased risk of both HIV acquisition and transmission [1]–[3]. Moreover, nonoxynol-9 (N-9), approved in the United States as a vaginal contraceptive, provides no protection against HIV or other sexually transmitted infections [4], [5] and frequent use was shown to be associated with an increased risk of HIV acquisition [6]. Thus, the development of safe and effective alternative contraceptives is a major global health priority.

The healthy human vagina in reproductive aged women is acidic, with a pH ranging from 3.5 to 4.5, primarily because of lactic acid and this environment inactivates sperm [7]. However, following sex, the pH is neutralized to at least 6.0 by semen (pH 7.2–8.2) to promote sperm survival. These observations provided the rationale for developing acid-buffering products as candidate multi-purpose agents that could serve as topical contraceptives and provide protection against acid-sensitive microbes.

Two acid-buffering products, BufferGel® (developed by ReProtect Limited Liability Company, Baltimore, MD) and Acidform (developed by the Program for Topical Prevention of Conception and Disease at Rush University, Chicago, IL) were formulated as vaginal gels. BufferGel® was safe and well tolerated in women [8]–[10] and reduced the prevalence of bacterial vaginosis (BV) when applied twice daily for 2 weeks [9]. However, it did not alter the risk of HIV infection in a large-scale effectiveness trial [10]. When combined with a diaphragm, BufferGel® was as effective as N-9 for contraception; the 6-month pregnancy rate per hundred women was 10.1% (95% confidence interval [CI] 7.1–13.1%) for BufferGel® and 12.3 (95% CI 7.7–16.9) for N-9 spermicide users [11]. However, no reduction in pregnancy rate was observed when BufferGel® was used alone and dosed pericoitally [10].

**Figure 1:**
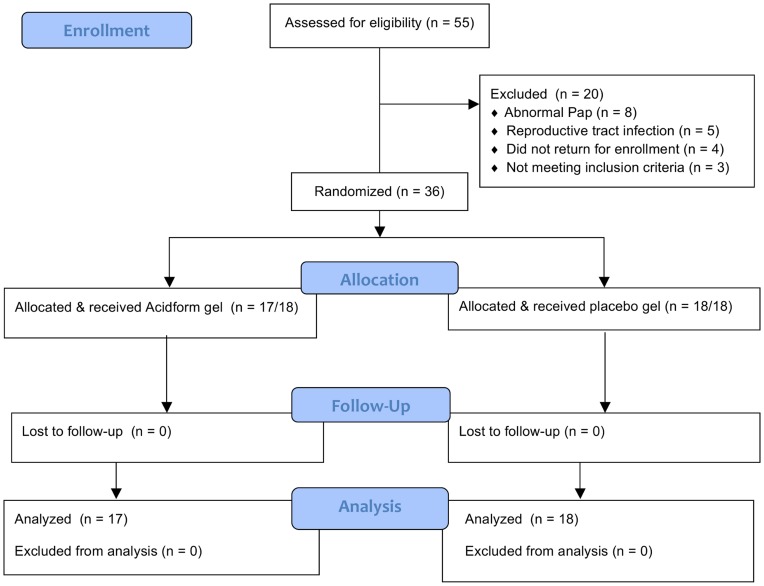
Trial profile.

Acidform is a bioadhesive formulation that contains lactic acid as a primary buffering agent. In contrast, the active ingredient in BufferGel® is the hydrogen ion, which is released from the buffering agent Carbopol 974 [8]. Acidform buffers twice the volume of semen to maintain a pH of 4.45 *in vitro*, is spermicidal [7] and is active against herpes simplex virus (HSV), *Chlamydia trachomatis* and *Neisseria gonorrhoeae* in animal studies [12]–[14]. A randomized, blinded, crossover study was conducted among 20 sexually active sterilized women to compare the spermicidal effect of Acidform to that of a commercial 2% N-9 product. Acidform or N-9 product administered 0–30 min precoitus or Acidform given 8–10 h precoitus significantly reduced the mean number of progressively motile sperm compared to control cycles (0.19, 0.07, 0.75 vs. 17.94, respectively, p<0.05, Wilcoxon signed-rank test) [15]. Acidform has been marketed as a personal lubricant (Amphora™ gel; Evofem Inc., San Diego, CA), and is currently being evaluated for contraceptive efficacy in a Phase III trial (ClinicalTrials.gov Identifier: NCT01306331).

Prior Phase I safety studies of Acidform gel alone or in combination with a diaphragm have been conducted. No symptoms or irritation were reported by 6 women who used Acidform gel once daily for 6 days [16]. In a second study, women were randomized to Acidform (n = 44) or K-Y Jelly (n = 28) and applied gel twice daily for 14 days [17]. Twenty-seven women in the Acidform group (61%) compared to 8 women in the K-Y Jelly group (29%) reported at least one symptom of genital irritation (odds ratio = 2.62, CI 1.30–5.31, p = 0.009). There was a trend towards more safety events in women who used Acidform with a diaphragm for six months compared to women who used a diaphragm with K-Y Jelly [18].

**Table 1:** Demographic data of recipients of Acidform and HEC placebo gel.

		Acidform Gel(N = 17)	Placebo Gel(N = 18)	p value
Age in years (mean ± standard deviation)		30.15±7.17	32.16±9.42	0.48
Race (number, %)*				0.34
	Black	6 (37.5%)	11 (61%)	
	White	4 (25%)	3 (16.7%)	
	Asian	0	1 (5.6%)	
	Mixed	6 (37.5%)	3 (16.7%)	
Ethnicity				0.72
	Hispanic	6 (35.3%)	5 (27.8%)	
	Non-Hispanic	11 (64.7%)	13 (72.2%)	
Level of Education				0.64
	Less than high school	0	1 (5.6)	
	High school/General education diploma	5 (29.4%)	3 (16.7%)	
	Some college	4 (23.5%)	6 (33.3%)	
	College	6 (35.3%)	4 (22.2%)	
	Graduate/Professional degree	2 (11.8%)	4 (22.2%)	
Number lifetime sex partners (median, range)		5 (1–34)	6 (0–100)	0.36
Reported history of anal sex		7 (41.2%)	7 (38.9%)	0.89
Current cigarette smoker		4 (23.5%)	4 (22.2%)	1.0
Tampon use		10 (58.8%)	12 (66.7%)	0.73
History of douching		5 (29.4%)	6 (33.3%)	1.0
Mean duration of menstrual cycle (days ± standard deviation)		28.6±2.12	28.1±1.76	0.42
Mean duration of menses (days ± standard deviation)		4.5±0.8	4.4±0.98	0.78
Current contraceptive method (number, %)				
	Male condoms	7 (41.2%)	9 (50%)	0.74
	Female condoms	1 (5.9%)	2 (11.1%)	1.0
	Tubal ligation	1 (5.9%)	0	0.49
	Intrauterine device	0	2 (11.1%)	0.49
	Withdrawal	0	2 (11.1%)	0.49
Prior history of vaginitis (number, %)				
	Candida vaginitis	10 (58.8%)	13 (72.2%)	0.49
	Bacterial vaginosis	2 (11.8%)	6 (33.3%)	0.23
Prior history of STI (number, %)				
	Trichomonas	0	2 (11.1%)	0.49
	Chlamydia	3 (17.6%)	3 (16.7%)	1.0
	Gonorrhea	1 (5.9%)	0	0.49
	Genital warts	1 (5.9%)	0	0.49
HSV seropositivity (number, %)				
	HSV-1 seropositive	13 (76.5%)	11 (61.1%)	0.47
	HSV-2 seropositive	3 (17.6%)	5 (27.8%)	0.47

* For one participant in the Acidform gel group, race is unknown.

The goal of the present study was to expand the safety assessment of Acidform gel with twice daily vaginal dosing over 14 days. Outcomes included symptoms, pelvic exam findings, concentrations of genital tract immune mediators, and quantification of antimicrobial activity of cervicovaginal secretions.

## Methods

### Ethics Statement

The protocol for this trial and supporting CONSORT checklist are available as supporting information; see Checklist S1 and Protocol S1. The study was conducted according to the Declaration of Helsinki and was approved by the Albert Einstein College of Medicine Institutional Review Board (IRB) and the NIAID Division of AIDS Prevention Science Review Committee. All study participants provided written informed consent.

**Table 2:** Adverse events related to Acidform and HEC placebo gel.

Participant Number	Gel Received	Adverse Event	Number of Episodes	Duration
1	Acidform	Vaginal Burning	3	1 min
4	Acidform	Vaginal Burning	2	5 min, 20 min
5	Acidform	Vulvar Itching	12	20 min
5	Acidform	Vulvar Itching	5	5 min
5	Acidform	Vulvar Erythema	1	3 days
9	Acidform	Abdominal Cramping	1	15 min
11	Acidform	Vulvar Dryness	1	2 days
13	Acidform	Vulvar Burning	3	30 min
13	Acidform	Vulvar Burning	1	10 min
13	Acidform	Vulvar Itching	1	30 min
13	Acidform	Vulvar Erythema	1	6 days
13	Acidform	Vulvar Abrasion	1	3 days
16	Acidform	Abdominal Cramping	1	60 min
16	Acidform	Vaginal Bleeding	1	13 days
17	Acidform	Abdominal Cramping	1	6.5 hours
18	Acidform	Vaginitis	1	4 days
22	Acidform	Vulvar Itching	2	10 min, 5 min
26	Acidform	Vulvar Itching	1	15 min
3	Placebo	Vulvar Itching	1	10 min
14	Placebo	Vaginal Itching	2	5 min

### Participants

Thirty-five healthy women between the ages of 18 and 50 years were recruited from the New York metropolitan area between February 2009 and December 2010. Inclusion criteria included regular menstrual cycles and willingness to abstain from sex for the duration of the study. Participants were excluded for pregnancy, breastfeeding, menopause, HIV infection, reproductive or urinary tract infection, bacterial vaginosis (BV), intermenstrual bleeding, abnormal Pap test, use of hormonal contraception during the study or in the previous two months, and antibiotic use in the week prior to enrollment.

**Figure 2:**
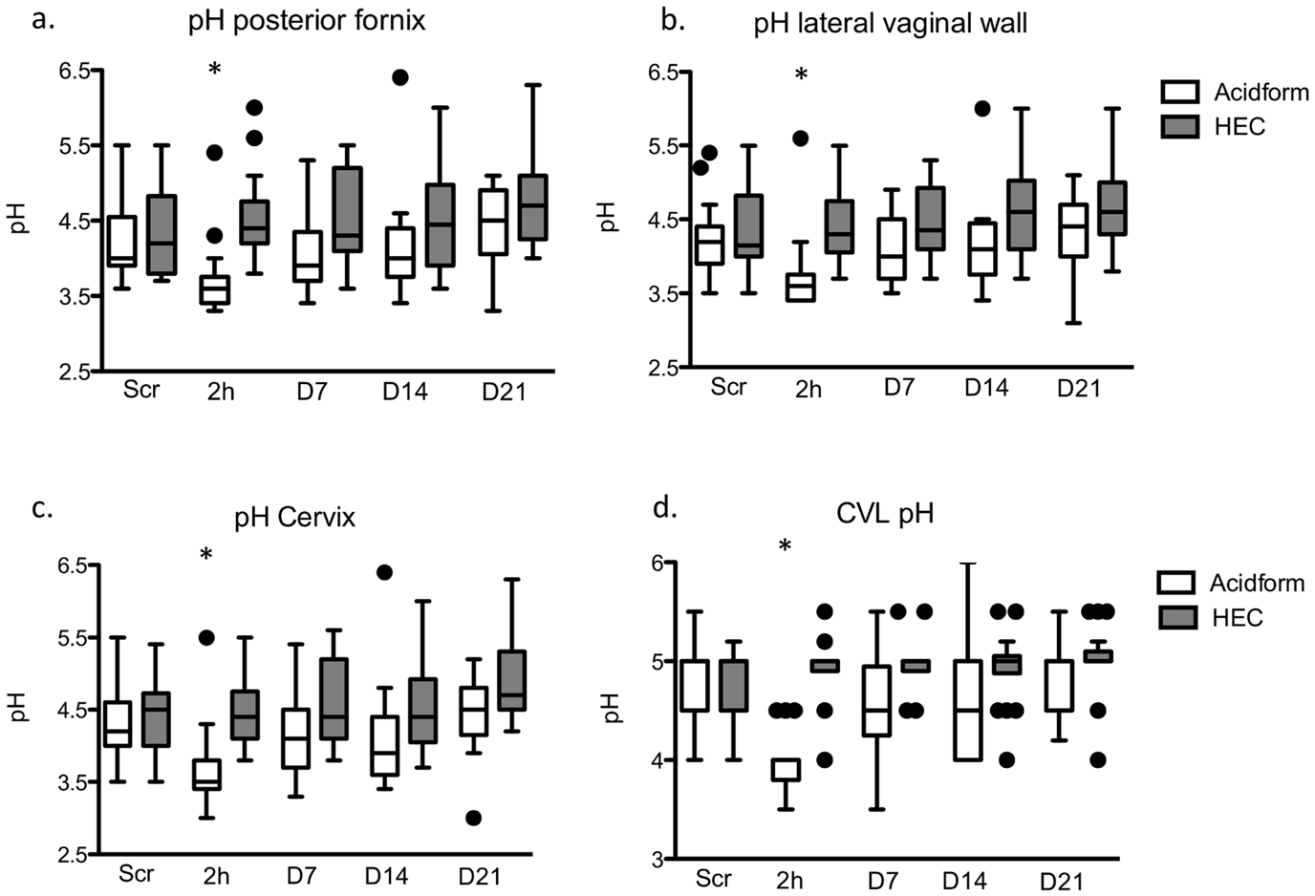
The pH of the vagina and cervix was significantly lower 2 hours after application of Acidform compared to HEC placebo gel. Box-and-whisker plots showing the pH of the posterior fornix (a), lateral vaginal wall (b), cervix (c) and CVL (d) obtained at screening (Scr), 2 hours (2 h) and at Days 7, 14 and 21 after insertion of Acidform (white) or HEC placebo gel (gray). The line indicates the median values and the circles are outliers. The asterisks denote a significant difference between the Acidform and HEC placebo group.

At screening, participants had urine collected for microscopy, culture and a pregnancy test. A gynecological examination was performed for detection of BV (wet preparation with Amsel clinical criteria), *Trichomonas vaginalis* (wet preparation), *Candida* species (KOH prep), and semen using an antibody immunoassay that detects p30, a glycoprotein produced by the prostate (Abacus Diagnostics, West Hills, CA). The pH was measured using a stainless steel sensor pH probe placed at the lateral vaginal wall, posterior fornix and cervix (ISFET PH77 Probe Hach Company, Loveland, CO). CVL was performed by washing the cervix and posterior fornix with 10 mL of normal saline (pH∼5.0). A Pap test was collected, and the presence of *Neisseria gonorrhoeae* and *Chlamydia trachomatis* infection was determined by nucleic acid amplification testing of endocervical swabs (Gen-Probe, Inc., San Diego, CA). Blood was collected for HIV ELISA, syphilis (rapid plasma reagin test), pregnancy, and serotype specific antibodies for HSV-1 and HSV-2 (HerpeSelect, Focus Diagnostics, Cypress, CA).

The enrollment visit (Day 0) was completed within 45 days of screening and 2–6 days after cessation of menstrual bleeding to allow ample time for dosing prior to anticipated onset of subsequent menses. Tests for bacterial vaginosis, *T. vaginalis*, *Candida* species, pH and semen detection were repeated. A swab of the lateral vaginal wall was collected to assess changes in vaginal bacterial populations. Eligible participants were then randomized 1∶1 to receive Acidform or hydroxyethylcellulose (HEC) gel; the randomization was computer generated by the pharmacist. The gels differed in color, which precluded randomization in a double-blind fashion. However, study participants and laboratory personnel were not informed of the random assignments. The first dose of Acidform or HEC was administered intravaginally by a study clinician. Two hours post-insertion, the pH was measured and CVL was performed. Participants were instructed to apply a dose twice daily, preferably in the morning and at bedtime and to not insert gel when study visits were scheduled until after the visit. The participants were provided with a diary to record usage and symptoms.

**Figure 3:**
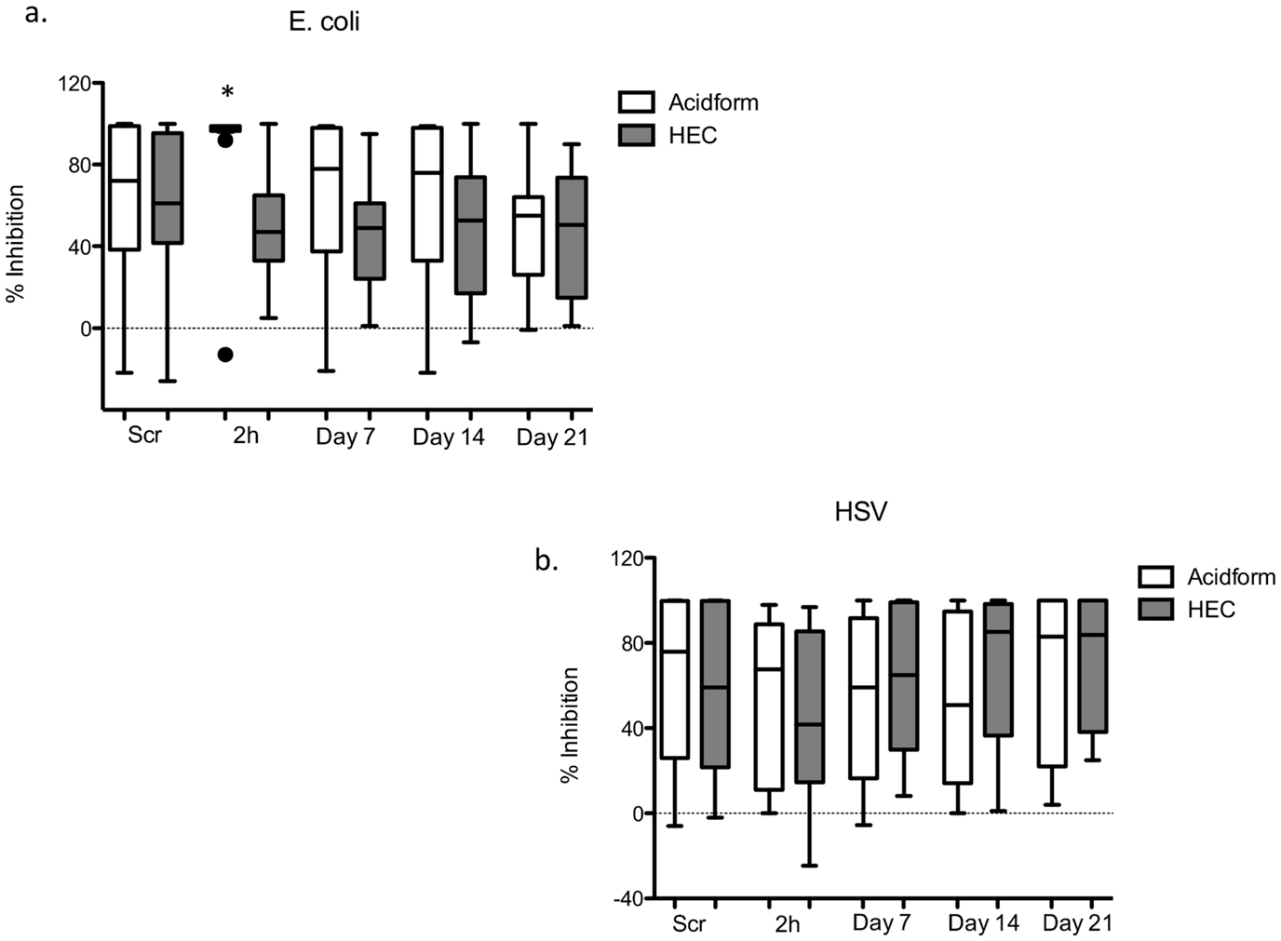
The bactericidal activity of genital tract secretions against *E. coli* was significantly greater 2 hours after application of Acidform compared to HEC placebo gel. Box-and-whisker plots showing the percent inhibition of *E. coli* (a) and HSV-2 plaque formation (b) in CVL samples collected at screening (Scr), 2 hours (2 h) and at Days 7, 14 and 21 after insertion of Acidform (white) or HEC placebo gel (gray). The line indicates the median values and the circles are outliers. The asterisk denotes a significant difference between the Acidform and HEC placebo group.

Subsequent study visits were conducted on Days 7 (range 5–9), 14 (range 14–16) and 21 (range 17–21). A speculum exam, wet mount microscopy, semen test, pH measurement and CVL were performed at each visit. At every visit, used and unused applicators were counted. Adverse event (AE) data were collected at each study visit and graded according to the NIH Division of AIDS Table for Grading the Severity of Adverse Events [19]. A swab of the lateral vaginal wall was collected on Day 14.

### Study Drugs

Acidform is a viscous, off-white gel containing three acidic compounds (lactic acid, citric acid and potassium bitartrate), a preservative (benzoic acid), two polymer thickeners (alginic acid and xantham gum), a humectant (glycerin), sodium hydroxide and water (pH 3.55). The placebo for this study is HEC gel, which is a water-based formulation that contains HEC, sodium chloride, sorbic acid and sodium hydroxide (pH 4.4) [20]. Both Acidform and HEC are applied with a single high-density polyethylene (HDPE) applicator capable of administering a 5 g (equal to 5 mL) dose of gel. Evofem, Inc. (formerly Instead Healthcare, LLC San Diego, CA) provided Acidform lubricant, HEC gel and HDPE applicators for this study.

### CVL Samples

CVL specimens were transported to the laboratory on ice and were clarified by centrifugation at 700 g for 10 minutes at 4°C. Supernatants were divided into aliquots and stored at −80°C. The protein concentration (Pierce Micro BCA) and pH (ColorpHast, pH 2–9, EMD Chemicals) of CVL samples were determined.

**Table 3:** Summary of concentrations of immune mediators and comparison of changes between the Acidform and HEC placebo groups.

Immune Mediators	Geometric Mean for Screening	Geometric Mean for2 hours	Geometric Meanfor Day 7	Geometric Meanfor Day 14	Geometric Meanfor Day 21	Estimated MeanDrug Effect	P-value time effect^a^	P-value drug effect
Total Protein (µg/ml)	280.2	165.3	164.7	189.7	251.3	−12.3	<0.01	0.62
Lysozyme (ng/ml)^b^	251.8	89.8	265.6	182.1	348.0	−30.5	<0.01	0.74
Lactoferrin (ng/ml)	1125.52	289.5	567.8	518.4	1131.3	−603.9	<0.01	0.04
HNP 1–3 (pg/ml)^c^	34637.8	14816.6	19285.3	31984.3	65336.1	−3354.0	<0.01	0.82
SLPI (pg/ml)^d^	219915.8	43761.1	88929.6	84897.5	88627.7	−36836.8	<0.01	0.21
IgG (ng/ml)	4929.0	3282.0	3490.5	3741.7	8950.8	−3188.8	<0.01	0.06
IgA (ng/ml)	981.4	256.9	407.3	603.3	1279.9	−363.8	<0.01	0.05
IL-1α (pg/ml)	58.6	7.7	26.0	31.3	55.4	−10.6	<0.01	0.35
IL-1ra (pg/ml)	6934.1	1825.1	3908.5	5201.0	9247.4	−3845.3	<0.01	<0.01
IL-1β (pg/ml)	4.2	1.1	3.6	2.8	17.8	−1.1	<0.01	0.60
IL-6 (pg/ml)	7.3	4.3	3.2	5.2	17.3	−3.7	<0.01	0.13
IL-8 (pg/ml)	482.7	90.6	169.9	183.5	883.9	−202.0	<0.01	0.08
IFN-γ (pg/ml)	1.5	0.5	1.0	0.9	1.4	−0.3	<0.01	0.28
MIP-1α (pg/ml)	LLOD: n = 9	n = 12	n = 8	n = 6	n = 2	n/a	<0.01	0.64
	<Q3: n = 17	n = 20	n = 22	n = 22	n = 19			
	≥Q3: n = 9	n = 2	n = 4	n = 7	n = 12			
MIP-1β (pg/ml)	LLOD: n = 13	n = 13	n = 13	n = 9	n = 4	n/a	<0.01	0.15
	<Q3: n = 16	n = 18	n = 18	n = 19	n = 18			
	≥Q3: n = 6	n = 3	n = 3	n = 7	n = 11			
RANTES (pg/ml)	LLOD: n = 9	n = 15	n = 16	n = 15	n = 4	n/a	<0.01	0.55
	<Q3: n = 21	n = 15	n = 16	n = 12	n = 20			
	≥Q3: n = 5	n = 4	n = 2	n = 8	n = 9			

Abbreviations: LLOD, lower limit of detection; Q3, 3^rd^ quartile; n/a, not applicable.

a As there was no significant interaction between treatment group and time, p-values <0.05 indicate that immune mediators changed over time, independent of whether participants applied Acidform or HEC placebo gel.

b Two values were assigned the highest standard multiplied by the dilution.

c Three values were assigned the highest standard multiplied by the dilution.

d One value was assigned the highest standard multiplied by the dilution.

### Antimicrobial Activity of CVL

The antimicrobial activity against HSV-2 and *E. coli* in CVL was measured as previously described [21]. For anti-HSV activity, Vero cells were infected with ∼50–200 pfu of HSV-2(G) mixed 1∶1 with each CVL or control buffer and plaques were counted after 48 hours. All samples were tested in duplicate in two independent experiments. To assess the bactericidal activity, *E. coli* (ATCC strain 4382627) was grown overnight to stationary phase and then 3 µl of bacteria (∼10^9^ cfu/ml) were mixed with 27 µl of CVL or control genital tract buffer (20 mmol/L potassium phosphate, 60 mmol/L sodium chloride, 0.2 mg/ml albumin, pH 4.5) and incubated at 37°C for two hours. The mixtures were further diluted in buffer (to yield 800–1000 colonies on control plates) and plated on agar enriched with trypticase soy broth. Colonies were counted using ImageQuant TL v2005 after an overnight incubation at 37°C. All samples were tested in duplicate and the percent inhibition was calculated relative to control wells.

### Measurement of Immune Mediators

Interleukin (IL)-1α, IL-1β, IL-6, IL-8, interferon (IFN)-γ, IFNa2, IL-1ra (IL-1 receptor antagonist), macrophage inflammatory protein (MIP)-1α, MIP-1β, and regulated upon activation, normal T-cell expressed and secreted (RANTES) were quantified in each CVL sample using a multiplex proteome array with beads from Chemicon International (Billerica, MA), measured using Luminex100 (Austin, TX) and analyzed using StarStation (Applied Cytometry Systems, Sacramento, CA). The levels of all other mediators were determined using commercial ELISA kits: secretory leukocyte protease inhibitor (SLPI) (R & D Systems), lactoferrin (Calbiochem, San Diego, CA), human neutrophil peptides 1–3 (HNP1–3) (HyCult Biotechnology, Uden, The Netherlands), IgG and IgA (CygnusTechnologies, Southport, NC) and lysozyme (Alpco Diagnostics, Salem, NH). The lower limit of detection (LLOD) for each assay in pg/ml was: HNP1–3, 156; SLPI, 25; IgG, 100; IgA, 150; lactoferrin, 1000; lysozyme 500; IL-1α, 3.5; IL-1β, 0.4; IL-6, 0.3; IL-8, 0.2; IFN-γ, 0.1; IFN-α2, 24.5; IL-1ra, 2.9; MIP-1α, 3.5; MIP-1β, 4.5; RANTES, 1.0. Sample values that were below the level of the lowest standard were set at the midpoint between zero and the LLOD and then multiplied by the dilution factor. Concentrations that were above the highest detection concentration were repeated at higher dilutions or, if insufficient sample was available, were assigned the value of the highest standard multiplied by the dilution.

### Dye Stain Assay (DSA) of Applicators to Assess Adherence

Each participant received 30 pre-filled individually packaged applicators of Acidform or HEC gel and was asked to return used and unused applicators at each study visit. Drug was dispensed from the returned unused applicators *ex vivo*, and all the applicators were then batched and stained with 0.05% FD&C Blue #1 granular food dye (Prime Ingredients INC, Saddlebrook, NJ) to detect whether the applicators had been inserted vaginally [22], [23]. Applicators inserted by study staff and unused applicators dispensed *ex vivo* by staff were included as positive and negative controls, respectively. Two independent observers scored the applicators as exposed or unexposed to vaginal mucus, and results were compared to subjects’ self-reports.

### Quantification of Vaginal Microbiota

DNA was extracted from stored vaginal swabs as previously described, with one extraction control for every 12 swabs. [24]. Human 18S rRNA gene polymerase chain reaction (PCR) was performed on all extracted DNA samples to ensure contact with vaginal mucosa during sampling and the presence of amplifiable DNA; amplification control PCR targeting a jellyfish aequorin gene was used to exclude the presence of PCR inhibitors [25]. Quantitative PCR (qPCR) assays utilizing primers and probes specific to each bacterium’s 16S rRNA gene were then used to quantify concentrations of key vaginal bacteria associated with health (*Lactobacillus crispatus* and *Lactobacillus jensenii*) and BV (*Gardnerella vaginalis*, *Megasphaera*-like bacterium (type 1 & type 2), and Clostridia-like bacterial vaginosis associated bacterium 2 (BVAB2)) [26]. Quantified bacterial levels were expressed as copies of bacterial DNA per vaginal swab.

### Study Outcome and Statistical Analysis

The primary objectives of this study were to examine the effect of Acidform or HEC placebo gel on antimicrobial activity and mediators of mucosal immunity. Secondary objectives were to assess the extent of acid buffering by Acidform after vaginal application and to evaluate a candidate biomarker of adherence. An exploratory objective was to evaluate changes in vaginal microbiota.

Demographic data at baseline between the two groups were compared using chi-squared or Fisher’s exact tests for categorical variables and using t-tests or Wilcoxon rank-sum tests for continuous variables. Differences in the rate of women with at least 1 AE between the two treatment groups were compared using Fisher’s exact test. Data on immune mediators were log transformed, where appropriate. Linear mixed models with a random intercept were used to examine changes in immune mediators during the study period and the effects of treatment on changes in immune mediators. For 3 immune mediators, (MIP-1α, MIP-1β, and RANTES), where more than 20% of immune mediators were below the limit of detection, immune mediators were converted to 3-level variables: 0 if immune mediators were undetectable, 1 if immune mediators were detectable but less than the 3^rd^ quartile of those with quantifiable levels, and 2 if immune mediators were at or above the 3^rd^ quartile. Generalized linear mixed models with a random intercept and a cumulative logit link function were used to examine changes in these 3 immune mediators during the study period. An interaction between treatment and time was included in the model to examine whether changes in immune mediators varied with treatment. Wilcoxon rank-sum tests were used to compare differences in pH, antimicrobial activity, and vaginal microbiota between the two treatment groups. Differences in *L. crispatus*, *L. jensenii*, *G. vaginalis* measured at enrollment and 14 days after gel use were compared using Wilcoxon signed-rank tests. Bonferroni adjustments were applied for post hoc comparisons between visits. Spearman’s correlation coefficients (SCC) were estimated to assess associations between antimicrobial activity and concentrations of immune mediators from screening samples. Agreement between two observers for differentiating applicators that were intravaginally inserted from those not intravaginally inserted was assessed using the kappa statistic. A kappa > = 0.75 indicated excellent agreement between the two observers. A kappa between 0.4 and 0.75 indicated fair to good agreement [27]. All statistical analyses were performed using SAS Version 9.2 (SAS Inc., Cary, NC, USA). All P values were two-tailed, with P<0.05 considered as statistically significant results.

**Table 4:** qPCR levels of bacteria from swabs collected at baseline (Day 0) and after 14 days of twice daily application of Acidform or HEC gel.

Gel group	Bacterium	# PCR + baseline	% PCR + baseline	Median DNA copies/swabat baseline	# PCR + Day 14	% PCR + Day 14	Median DNA copies/swab^a^ at Day 14
Acidform (n = 17)							
	*L. crispatus*	10	59	9.3×10^7^	12	71	5.9×10^6^
	*L. jensenii*	11	65	7.6×10^6^	12	71	1.4×10^7^
	*G. vaginalis*	10	59	1.3×10^6^	8	47	3.6×10^4^
	*Megasphaera*	1	6	4.6×10^7*^	1	6	1.8×10^7b^
	BVAB2	1	6	1.8×10^7*^	1	6	2.1×10^7b^
HEC (n = 18)							
	*L. crispatus*	8	44	5.9×10^7^	9	50	4.2×10^7^
	*L. jensenii*	9	50	6.8×10^6^	9	50	1.1×10^7^
	*G. vaginalis*	16	89	9.8×10^5^	13	72	4.4×10^6^
	*Megasphaera*	4	22	3.2×10^7^	3	17	1.4×10^6^
	BVAB2	4	22	1.2×10^6^	4	22	1.9×10^5^

a Median DNA copies/swab among participants with detectable DNA.

b DNA copies/swab for one participant.

### Sample Size

A total sample size of 36 (18 subjects in each arm) was selected *a priori* to allow 80% power to observe statistically significant equivalence of means if we assume that Acidform and HEC have no impact on anti-HSV activity and consider the mean difference of 1 standard deviation as the equivalence limit.

## Results

### Study Subjects

Fifty-five women were assessed for eligibility, and 35 were enrolled (Fig. 1). Study product expired prior to enrollment of the final participant. The majority of exclusions were due to an abnormal Pap test (n = 8) or reproductive tract infection (n = 5). All 35 participants completed the trial, which included 17 in the Acidform and 18 in the placebo group. The mean age of the enrolled women was 31.2 years and there were no differences in race, ethnicity, education or other demographic characteristics between participants in the Acidform compared to the placebo group (Table 1).

### Tolerance of Acidform Gel

There were 51 AEs among 18 women. Forty-two of the AEs, which occurred among 13 women (11 in the Acidform and 2 in the HEC group), were possibly or probably related to the study products (Table 2). Eleven of 17 (65%) women who received Acidform had at least 1 AE compared with 2 of 18 (11%) who received HEC placebo gel (p = 0.002). The most commonly reported AEs were genital tract itching and burning. Two subjects developed vulvar erythema that was temporally linked to Acidform application. All product related AEs were graded as mild and most occurred immediately following gel application.

### Effects of Acidform on pH and on Antimicrobial Activity of Genital Secretions

The median pH at the cervix, lateral vaginal wall, posterior fornix and CVL was significantly lower 2 hours after application of Acidform compared to the placebo group (p<0.01) (Fig. 2). There were no significant differences between the groups in pH at any location at screening, Day 7, 14 or 21, although there was a trend towards lower pH at all locations on days 7 and 14 in the Acidform group.

The activity of female genital secretions collected by CVL against *E. coli* and HSV was measured *ex vivo* at each time-point. The median percent inhibition of *E. coli* was 72 [interquartile range (IQR) 36–99] and 61 [42–95] at screening in the Acidform and HEC groups, respectively. Consistent with a lower pH 2 hours after Acidform gel application, we found that the bactericidal activity of CVL against *E. coli*, which are susceptible to lactic acid, was significantly greater and less variable 2 hours after initial application of Acidform compared to placebo (98 [96.5–99] for Acidform vs. 47 [33–65] for HEC, p<0.01, Fig. 3). However, bactericidal activity did not differ significantly between the drug and placebo group at any of the other time-points, which is consistent with the absence of any significant differences in pH at the other time-points (Figs. 2 and 3). The median percent inhibition of HSV-2 plaque formation was 76 [26–100] and 59 [21.5–10] at screening in the Acidform and HEC arms, respectively. There were no statistically significant differences in anti-HSV activity between the Acidform and placebo group after initial or repeated gel application (Fig. 3).

### Effects of Acidform on Inflammatory Mediators and Host Protective Factors

The concentrations of the majority of cytokines, chemokines, and antimicrobial proteins decreased 7 and 14 days after vaginal gel use, independent of whether participants applied Acidform or HEC placebo gel, because there was no significant interaction between treatment group and time (Table 3). These time-points corresponded with menstrual cycle days 13–18 and 20–25, respectively, and likely reflect the physiological nadir [28]–[30]. There was a statistically significant drug effect observed for lactoferrin (p = 0.04) and IL-1ra (p<0.01) (Table 3). In the Acidform group, lactoferrin and IL-1ra concentrations in CVL were significantly lower compared to the HEC group. The estimated mean drug effects for lactoferrin and IL-1ra were −603.9 ng/ml and -3845.3 pg/ml, respectively (Table 3). No significant drug effect was observed for the other cytokines, chemokines and host protective factors. However, there was a trend towards a reduction in CVL concentrations of IgG (p = 0.06), IgA (p = 0.05) and IL-8 (p = 0.08) in women who applied Acidform gel.

### Correlation of Antimicrobial Activity with Mucosal Immune Mediators and Vaginal pH

The *E. coli* bactericidal activity correlated negatively with the pH of the vaginal wall (ρ = -0.42, p = 0.01) and positively with total protein (ρ = 0.67, p<0.0001), but not other immune mediators. In contrast, the anti-HSV activity correlated modestly and significantly with concentrations of IL-1α (r = 0.55, p = 0.0005), IL-1β (r = 0.45, p = 0.007), IL-8 (r = 0.52, p = 0.001), HNP1–3 (r = 0.42, p = 0.01), lysozyme (r = 0.36, p = 0.03), and IgA (r = 0.34, p = 0.04), but not with vaginal pH (r = −0.11, p = 0.54). These correlations are consistent with results obtained in other studies [21], [31], [32].

### Effects of Acidform on Vaginal Microbiota

The number of women with *L. crispatus* or *L. jensenii* detected at enrollment did not differ significantly between the two groups and represented 51% and 57%, respectively. There were no significant changes in the number of women with detectable *L. crispatus or L. jensenii* or in the concentrations of bacteria recovered by PCR in the Acidform or HEC group following 14 days of twice daily gel use (Table 4). However, there was a trend towards a decrease in the concentration of *G. vaginalis* following repeated application of Acidform from a median of 1.3×10^6^ to 3.6×10^4^ DNA copies/swab (p = 0.083). There were no cases of BV diagnosed by Amsel criteria throughout the study period. Five women had high levels of either *Megasphaera* or BVAB2 detected at the enrollment visit and 14 days later, suggesting that these women may have had unrecognized BV not revealed by Amsel clinical criteria.

### Measurement of Adherence by Applicator Staining

Subjects were instructed to return used and unused applicators. The returned applicators were stained within four months along with positive (applicators inserted by study staff) and negative (unused applicators that had been dispensed *ex vivo*) controls. A total of 1012 of 1050 (96%) polyethylene applicators were returned, including 935 that participants reported had been intra-vaginally applied and 77 unopened pre-filled applicators. Four participants reported missing 1 dose, 2 reported missing 3 doses and 2 reported missing 5 doses of gel.

Both observers correctly identified 29 of the 30 applicators inserted by study staff as positive and scored 87% and 96%, respectively, of the returned applicators that were reported to have been used as positive. The two observers identified 40/60 (66%) and 58/60 (96%) of the negative controls as negative and 55/77 (71%) and 58/77 (75%), respectively, of the returned unused applicators as negative. There was good agreement between the observers, as demonstrated by a kappa of 0.64, 95% confidence interval (0.58, 0.69). The sensitivity of the DSA for the two observers was 87% and 84%, respectively and the specificity was 69% and 85%, respectively. The positive predictive value was 95% and 97%, respectively and the negative predictive value was 44% and 43%, respectively.

## Discussion

Acidform was found to be more irritating than HEC placebo gel, with a greater proportion of mild genital symptoms. A similar increased rate of mild AEs was observed in an earlier study when Acidform was compared to K-Y Jelly [17]. Despite the findings of mild irritation associated with Acidform use, we observed no increase in pro-inflammatory cytokines or chemokines, although there was a significant decrease in the concentration of the anti-inflammatory protein, IL-1ra in CVL obtained from participants who applied Aciform compared to HEC gel. Whether the mild irritation and the decrease in IL-1ra observed in this study portend a risk for mucosal inflammation with more prolonged exposure to Acidform requires further study.

The median pH of the cervix and vagina 2 hours after a clinician administered Acidform gel was 3.5–3.7, which was associated with increased *E. coli* bactericidal activity, and is low enough to completely immobilize spermatozoa if the pH is maintained following coitus. However, no significant differences in bactericidal activity or pH at the lateral vaginal wall, posterior fornix or cervix were observed at any other time-point, which suggests that the acid-buffering effects do not persist. The average time elapsed between gel dosing and CVL sampling for the Day 7 and 14 study visits was 13 and 16 hours, respectively. These findings suggest that Acidform may be effective as a topical contraceptive and may prevent vaginal *E. coli* colonization, which has been observed after sexual intercourse with and without a condom [33]. We recently demonstrated an inverse correlation between vaginal *E. coli* colonization and bactericidal activity of CVL, indicating that the *ex vivo* activity may translate to protection against colonization [34]. However, the transient nature of the responses observed in the current study suggests that the product would have to be applied shortly prior to sex to be effective. The need to apply gel shortly prior to intercourse, the brief duration of activity, and the potential to develop genital irritation may limit adherence, acceptability and efficacy of Acidform gel as a topical contraceptive.

Despite potent antiviral activity in a mouse model [12], there was no significant increase in anti-HSV activity of CVL following Acidform gel use. These findings may reflect the pH of CVL (mean of 4 at 2 hours and >4.5 at all other visits), which was likely not sufficient to inhibit HSV. We previously demonstrated that HSV inactivation *in vitro* is rapid and substantial at pH 3.5, but less effective at pH of 4.5 [12]. The anti-HSV activity of CVL correlates with concentrations of several immune mediators including lactoferrin, IL-8 and IgA; each of these was lower following Acidform use and this may have also contributed to the absence of any increase in anti-HSV activity. Notably, lactoferrin, an anti-bacterial glycoprotein produced by epithelial cells and neutrophils has been shown to inhibit HSV *in vitro* [35]. While we observed no loss in the anti-HSV activity of CVL in this study, the lower levels of lactoferrin and other immune mediators following Acidform gel application suggest that more prolonged or frequent exposure could potentially interfere with mucosal defense. Further studies are needed to determine the clinical significance of these findings.

The healthy human vaginal microbiome is dominated by *Lactobacillus* species at high concentrations, including *L. crispatus* and *L. jensenii* [36], which maintain acidic vaginal pH by producing lactic acid. BV occurs when these beneficial vaginal lactobacilli are replaced by overgrowth of commensal vaginal anaerobes [36], [37]. In a previous Phase I study, there was no significant increase in hydrogen peroxide-producing lactobacilli following repeated vaginal application of Acidform gel, as measured by semiquantitative vaginal cultures [17]. In the present study, qPCR was utilized to assess the impact of Acidform gel on absolute quantities of protective lactobacilli and BV-associated organisms. There was no increase in the concentrations of beneficial lactobacilli following use of Acidform gel. Notably, there was a decrease in the amount of *G. vaginalis* recovered following Acidform, but not HEC gel application, on Day 14. *Gardnerella vaginalis* is almost always present in the vaginas of women with BV, but is also found in 70% of women without BV [26]. These findings suggest that the product may provide at least some colonization resistance against *G. vaginalis*, although further studies with sexually active women are needed to determine the potential role Acidform may play in promoting a healthy microbiome. Future studies should include more comprehensive studies of the effects of Acidform on the vaginal microbiota.

This is the first study to apply the DSA to polyethylene applicators used to insert gel more than once daily. The DSA has been previously studied with methylcellulose, HEC, Carraguard, and PRO 2000 gels inserted once daily from low-density polyethylene (LDPE) applicators, with sensitivity and specificity of >90% [22], [38], [39]. The sensitivity of the assay was decreased when studied with VivaGel gel applied twice daily from polypropylene applicators [23], [39]. The difference may have been due to gel remaining in the vagina from the previous insertion. Alternatively, the sensitivity and specificity of the DSA may vary with the type of applicator used (polyethylene vs. polypropylene). In a recent study of tenofovir gel applied once daily with polypropylene applicators [21], the sensitivity of the DSA was >90% but the specificity was <70%, suggesting that staining of polypropylene applicators may not be as effective as that of polyethylene. In the current study of twice daily dosing of Acidform or HEC from polyethylene applicators, the sensitivity of the DSA for two observers was 87% and 84%, respectively and the specificity was 69% and 85%, respectively. These results suggest that the DSA may not be an effective method to assess applicator use when gel is administered more than once daily, despite the type of applicator used. Important limitations of the DSA are the differences in technique used by different groups and the high degree of interobserver variability [21], [23], [39], indicating the need for standardization and training to reduce subjectivity. These findings also highlight the need for better markers of adherence. A recent study suggests that direct inspection of polypropylene applicators under ultraviolet light may provide a reliable assessment of adherence [40].

In summary, Acidform gel was associated with a decrease in pH and an increase in *E. coli* bactericidal activity 2 hours after gel application as well as a decrease in the concentration of *G. vaginalis* recovered on Day 14, suggesting that it may promote a healthier vaginal microbial environment. However, twice daily application of Acidform was associated with mild irritation and lower CVL levels of several immune mediators compared to HEC placebo gel. Determination of the clinical significance of these findings requires additional safety studies of this candidate non-hormonal contraceptive with sexually active populations and more prolonged product exposure.

## Supporting Information

Checklist S1 **CONSORT Checklist.**
(DOC)

Protocol S1 **Trial Protocol.**
(DOC)
