# Supplementary material for: Phase I Randomized Safety Study of Twice Daily Dosing of Acidform Vaginal Gel: Candidate Antimicrobial Contraceptive
Source: PLoS One. 2012 Oct 8;7(10):e46901. doi: 10.1371/journal.pone.0046901 (PMC3466198; doi:10.1371/journal.pone.0046901)
Supplement: Protocol S1 — Trial Protocol. (DOC) [file pone.0046901.s002.doc]

# AF 020

**Safety of Acidform Lubricant (Amphora) in Women at Low Risk for HIV–1 Infection**

# Sponsored by:

# Albert Einstein College of Medicine

# Co-sponsored by:

Division of AIDS, National Institute of Allergy and Infectious Diseases

National Institutes of Health

U01 AI069551

**Pharmaceutical Support:**

Instead Healthcare, LLC

**Principal Investigator:** Marla Keller, M.D.

Signature Page

The signature below constitutes the approval of this protocol and the attachments, and provides the necessary assurances that this trial will be conducted according to all stipulations of the protocol, including all statements regarding confidentiality, and according to local legal and regulatory requirements and applicable U.S. Federal regulations and ICH guidelines.

Principal Investigator: Marla Keller, M.D.

Signed: ________________________________ Date: _____________

TABLE OF CONTENTS

*Abbreviations and Acronyms 5*

*Protocol Summary 6*

1.0 Key Roles 7

2.0 Introduction 10 2.1 Background and Rationale 10

2.2 *Acidform Lubricant and Hydroxyethylcellulose Gel Prior Research* *13*

2.3 Potential Risks and Benefits 15

3.0 Objectives 16

3.1 Primary Objective 16

3.2 Secondary Objective 16

*3.3 Exploratory Objectives 16*

4.0 Study Design 16

4.1 Laboratory Methods 18

5.0 Study Population 20

5.1 Participant Inclusion Criteria 20

5.2 Participant Exclusion Criteria 21

6.0 Study Product/Intervention(s) 22

6.1 Regimen 22

6.2 Study Product Formulation and Preparation 22

6.3 Study Product Supply, Packaging and Accountability 23

6.4 Assessment of Participant Adherence with Study Product/Intervention(s) 23

6.5 Concomitant Medications 24

7.0 Study Procedures/Evaluations 24

7.1 Clinical Evaluations and Procedures 24

7.2 Interim Contacts and Visits (ad hoc) 30

7.3 Specimen Preparation, Handling and Shipping 30

7.4 Biohazard Containment 30

8.0 Assessment of Safety 30

8.1 Adverse Events 30

8.2 Local regulatory requirements 31

8.3 Social harms reporting 31

9.0 Clinical Management 32

9.1 Toxicity Management 32

9.2 Other Disease Events 32

9.3 Pregnancy 32

9.4 Breastfeeding 32

9.5 Criteria for Discontinuation 32

9.6 Criteria for Permanent Treatment Discontinuation 33

9.7 Criteria for Premature Study Discontinuation 33

10.0 Statistical Considerations 33

10.1 Overview and Statistical Design Issues 33

10.2 Sample Size Considerations 34

10.3 Enrollment/Randomization/Blinding Procedures 35

10.4 Participant Enrollment and Follow-Up 35

10.5 Data and Safety Monitoring 35

10.6 Safety Review 36

11.0 Data Handling and Recordkeeping 36

11.1 Data Management Responsibilities 36

11.2 Source Documents and Access to Source Data/Documents 37

12.0 Human Subjects Protection 37

12.1 Institutional Review Board 37

12.2 Informed Consent Process 37

12.3 Participant Confidentiality 37

12.4 Study Discontinuation 38

13.0 Publication Policy 39

14.0 References 40

Appendices 42

Appendix I Schedule of assessments 42

Appendix II Laboratory Evaluations 43

Appendix III Instructions for Using Acidform Lubricant or HEC gel 44

Appendix IV Outcomes, Diagnostics and Follow-Up Procedures 45

ABBREVIATIONS AND ACRONYMS

AE adverse event

AECOM Albert Einstein College of Medicine

AIDS acquired immune deficiency syndrome

BV bacterial vaginosis

CDC Centers for Disease Control and Prevention

CDRH Center for Devices and Radiological Health

CFU colony forming units

CS cellulose sulfate

CVL cervicovaginal lavage

DAIDS Division of AIDS

EIA enzyme immunoassay

FACS fluorescent activated cell sorter

FDA (United States) Food and Drug Administration

FTA-ABS fluorescent treponemal antibody absorption

GCRC General Clinical Research Center

HBD human beta defensins

HCG human chorionic gonadotropin

HEC hydroxyethylcellulose

HIV-1 human immunodeficiency virus, type 1

HNP human neutrophil peptides

HPV human papillomavirus

HSV herpes simplex virus

IFN interferon

IgA immunoglobulin A

IgG immunoglobulin G

IL interleukin

IL-1ra interleukin-1 receptor antagonist

IND investigational new drug

IRB institutional review board

mAbs monoclonal antibodies

MMC Montefiore Medical Center

NIH National Institutes of Health

N-9 nonoxynol-9

OHRP Office for Human Research Protections

PAR Program announcement identifying location of peer review

PCR polymerase chain reaction

PBS phosphate buffered saline

PMPA phosphonylmethoxy propyl adenine monohydrate

PSA prostate-specific antigen

qRT-PCR quantitative real-time polymerase chain reaction

RANTES regulated upon activation, normal T cell expressed and secreted

RPR rapid plasma reagin

SLPI secretory leukocyte protease inhibitor

sd standard deviation

STI sexually transmitted infections

TER transepithelial resistance

TPHA treponemal pallidum hemagglutination

TNF tumor necrosis factor

UADE unanticipated adverse device effec

# PROTOCOL SUMMARY

# Full Title:

# Safety of Acidform Lubricant (Amphora) in Women at Low Risk for HIV–1 Infection

# Clinical Phase:

Phase I

Principal Investigator:

Marla Keller, MD

Sample Size:

Thirty-six participants will be enrolled in this study

Study Population:

Sexually abstinent, HIV uninfected women with a normal lower genital tract between the ages of 18 and 50

Participating Sites:

Albert Einstein College of Medicine and Montefiore Medical Center

Study Design:

This is a randomized, open-label study comparing the safety of Acidform lubricant to hydroxyethylcellulose (HEC) gel used twice daily for 14 consecutive days.

Study Duration:

Accrual will require approximately 18 calendar months. Each participant will be followed for 3 weeks. Study participants will remain in this study for approximately two months.

Objectives:

To compare the safety of Acidform lubricant to HEC gel by assessing the genital tract mucosal response to 14 days of twice daily application by measuring endogenous antimicrobial activity and levels of defensins, cytokines, and chemokines in cervicovaginal lavage (CVL).

Exploratory objectives include: (1) determining whether intravaginal application of Acidform lubricant or HEC results in changes in epithelial integrity by collecting cervical biopsies, (2) examining changes in vaginal flora following application of Acidform lubricant or HEC gel and (3) examining the antiviral activity in CVL collected 2 hours following a single application of Acidform lubricant or HEC gel using a spiking strategy.

**1.0** **KEY ROLES**:

# Principal Investigator: Marla Keller, M.D.

# Associate Professor

# Albert Einstein College of Medicine

# 1300 Morris Park Avenue

# Bronx, NY 10461

# Tel: (718) 430-3240

# Fax: (718) 430-8879

mkeller@aecom.yu.edu

# Co-Investigators: Betsy Herold, M.D.

# Professor

# Albert Einstein College of Medicine

# 1300 Morris Park Avenue

# Bronx, NY 10461

# Tel: (718) 430-2974

# Fax: (718) 430-8627

# bherold@aecom.yu.edu

Mark Einstein, M.D., M.S.

Assistant Professor

Albert Einstein College of Medicine

1695 Eastchester Road, Room 601

Bronx, NY 10461

Tel: (718) 405-8082

Fax: (718) 405-8087

meinstei@montefiore.org

Rodney Wright, M.D.

Assistant Professor

Albert Einstein College of medicine

1825 Eastchester Road, 7N

Bronx, NY 10461

Tel: (718) 904-2767

Fax: (718) 904-2799

rwright@montefiore.org

Clinical Research Coordinators: Julie Petrie, M.P.H.

Albert Einstein College of Medicine

1300 Morris Park Avenue

Bronx, NY 10461

Tel: (718) 430-3253

Fax: (718) 430-8879

jpetrie@aecom.yu.edu

Tara Ford, P.A.

Albert Einstein College of Medicine

1300 Morris Park Avenue

Bronx, NY 10461

Tel: (718) 430-3061

Fax: (718) 430-8879

tford@aecom.yu.edu

Pharmaceutical Support: Joseph D. Pike

Instead Healthcare, LLC

4275 Executive Square, Suite 440

La Jolla, CA 92037

Tel: (858) 550-1900, Ext. 103

Fax: (858) 550-0119

joepike@akcesspacific.com

Safety Committee: Kathryn Anastos, M.D.

Professor

Montefiore Medical Center

3311 Bainbridge Avenue

Bronx, NY 10467

Phone: (718) 515-2593

Fax: (718) 547-0584

kanastos@verizon.net

Karen Beckerman, M.D.

Associate Professor

Albert Einstein College of Medicine

1825 Eastchester Road

Bronx, NY 10461

Tel: (718) 904-2767

Fax: (718) 904-2799

kbeckerm@montefiore.org

Program Officer: Jim A. Turpin, Ph.D.

Topical Microbicide Team

Microbicide Research Branch
Prevention Sciences Program
Division of AIDS
6700B Rockledge Drive, Rm. 5114

Bethesda, MD 20892-7628
Phone: (301) 451-2732

Fax: (301) 496-8530

jturpin@niaid.nih.gov

Medical Officer: Lydia Soto-Torres, M.D., M.P.H.

Medical Officer

Microbicide Research Branch
Prevention Sciences Program
Division of AIDS
6700B Rockledge Drive, Rm. 5138

Bethesda, MD 20892-7628
Phone: (301) 594-9705

Fax: (301) 435-651

lsoto-torres@niaid.nih.gov

Clinical Operations Support: Grace C. Chow

Prevention Research Branch

Prevention Sciences Program

Division of AIDS

6700B Rockledge Drive, Rm. 5119

Bethesda, MD 20892-7628

Phone: (301) 435-3747

Fax: (301) 402-3684

gchow@niaid.nih.gov

2.0 INTRODUCTION

## 2.1 Background and Rationale

## The Joint United Nations Programme on HIV/AIDS estimated that 33.2 million people were living with human immunodeficiency virus (HIV) by the end of 2007 (1). The majority of new infections are transmitted through heterosexual contact. As such, there is a clear need for new technologies to prevent the sexual transmission of HIV. Correct and consistent male condom use has been shown to prevent HIV transmission (2), but women often are unable to negotiate the use of condoms by their male partners.

Topical microbicides are products designed to prevent the sexual transmission of HIV and other disease pathogens (3). Potentially, they can be applied vaginally to prevent both acquisition and transmission. They also offer a female-controlled option in cases where male condom use cannot be negotiated. A critical gap in the preclinical and clinical development of candidate microbicides and formulations is the establishment of surrogate markers of safety and efficacy. Vaginal products may induce molecular alterations in the mucosal environment, including the loss of host defenses or the induction of inflammation, which are not reflected by colposcopic changes. These alterations could facilitate acquisition of infection or trigger HIV replication. This notion is supported by clinical trial failures with nonoxynol-9 (N-9) and more recently, with cellulose sulfate (CS). While N-9 has been extensively used as a spermicide and colposcopy studies suggested that N-9 gels were safe, a Phase III trial found that frequent use was associated with an increased risk of HIV acquisition and that it provided no protection against HIV or other sexually transmitted infections (STI) (4). Particularly concerning was the unanticipated finding of an increased risk of HIV in one of the two Phase III CS clinical trials, which resulted in premature closure of both studies (5, 6). This result was not predicted by the Phase I studies, which were conducted among hundreds of women and used colposcopic changes as a primary endpoint. These experiences highlight the need for modifications of microbicide safety studies.

Acidform gel received 510(k) clearance from the Food and Drug Administration (FDA) for use as a lubricant (# K033776) in June 2004 and has been licensed to Instead Healthcare, LLC for commercialization under the name Amphora. Acidform was originally developed by the Program for the Topical Prevention of Conception and Disease (TOPCAD) (Chicago, IL). Important attributes of this lubricant are its contraceptive and antimicrobial activity, which although unlikely to be sufficient alone to protect against HIV or other STI, indicate that Acidform could prove to be an optimal vehicle for formulating acid-stable candidate microbicides. Acidform has been shown to acidify ejaculate more effectively than BufferGel, Aci-Jel, K-Y Jelly and Conceptrol gel (7, 8). Additionally, *in vitro* studies demonstrate that both HIV and herpes simplex virus (HSV) are inactivated by acidic pH (9, 10). Specifically, we found that Acidform protected 80% of mice from genital herpes infection when HSV-2 was introduced in phosphate buffered saline (PBS) and 60% of mice when virus was introduced diluted in human seminal plasma (pH ~8.2) (10). To further understand the buffering capacity of Acidform, human genital tract secretions (pH 4.2) were mixed with an equal volume of Acidform (pH 3.5) and with increasing amounts of seminal plasma (pH 8.2) and the final pH determined. Acidform buffered a 1:1:1 mixture (genital tract secretions: Acidform: seminal plasma) to pH 3.5. However, as the volume of seminal plasma was increased, the acid-buffering properties were overcome. At a relative ratio of 1:1:4, the resulting pH was 6.0 (10). In addition, a recent study demonstrated only modest susceptibility of non-clade B HIV-1 isolates to acidic pH (9). All of the primary HIV-1 isolates tested retained infectivity after exposure to pH 4.5, with most retaining >50% infectivity for up to 30 minutes (9). These findings suggest that Acidform lubricant alone would provide only partial protection against HIV or HSV, but suggest that the gel could provide an excellent vehicle for formulation of other candidate microbicides, with the additional benefit of providing contraception.

The goal of this proposed non-network clinical trial, which is sponsored by the Integrated Preclinical/Clinical Program for HIV Topical Microbicides PAR 03-137 is to compare the genital tract mucosal response to 14 days of twice daily application of Acidform lubricant to HEC gel by measuring endogenous antimicrobial activity and levels of cytokines, chemokines, defensins and other protective proteins in cervicovaginal lavage (CVL). Exploratory objectives include determining whether intravaginal application of Acidform lubricant or HEC results in alterations in epithelial integrity, examining changes in vaginal flora and examining the the antiviral activity of Acidform lubricant by evaluating the ability of CVL collected 2 hours after a single application of Acidform or HEC to inhibit both HIV and HSV using a spiking strategy developed in our laboratory. These studies should help determine whether Acidform would serve as an appropriate vehicle for formulation of candidate acid-stable microbicides.

This study builds on our previous work in which we tested the effect of 14 days of daily application of 0.5% PRO 2000 gel, a naphthalene sulfonic acid polymer currently in Phase IIB/III clinical trials, on levels of inflammatory, anti-inflammatory and protective immune mediators and on the endogenous antimicrobial activity of cervicovaginal fluid (11). Cytokines and chemokines were lower and protective factors including IL-1 receptor antagonist (IL-1ra), immunoglobulins, secretory leukocyte protease inhibitor (SLPI) and human beta-defensin (HBD) 2 were reduced in CVL in the PRO 2000 compared to the placebo group on Days 7 and 14, but returned towards normal on Day 21 (7 days after the last exposure to drug). Importantly, the modest reduction in protective factors was not associated with any detectable loss in the endogenous antiviral (HIV or HSV) or antibacterial (*E. coli* or *Staphylococcal aureus*) activity in CVL samples. We hypothesize that inclusion of measurement of endogenous antimicrobial activity or levels of specific mediators in CVL in Phase I clinical studies may provide biomarkers predictive of safety.

We recently completed a pilot study to compare endogenous anti-HSV activity and levels of immune mediators in CVL from women who were naturally cycling (n=9) to women receiving hormonal contraception (n=7). We found that women receiving hormonal contraception had decreased intrinsic anti-HSV activity compared to cycling women. In our 14-day PRO 2000 study, women who were naturally cycling had lower levels of several mediators of mucosal immunity mid-cycle and during the proliferative phase relative to women using hormonal contraception (11). Given the limited data on the effect of reproductive hormones on mucosal immunity, we will limit enrollment to naturally cycling women to assess the impact of Acidform application on endogenous antimicrobial activity and mucosal immune mediators.

This study also builds on our previous work in which we tested the antiviral activity in CVL collected after a single application of 0.5% PRO 2000 gel or matched placebo using a spiking strategy (12). We found that CVL obtained 1 hour after a single application of 0.5% PRO 2000 gel significantly inhibited HIV and HSV infection *in vitro* at least 1000-fold, indicating that the drug is sufficiently bioavailable and retains substantial antiviral activity after intravaginal application. However, the antiviral activity was reduced if virus was introduced diluted in human seminal plasma (13). In the proposed study, we will measure the antiviral activity provided by Acidform by collecting CVL post-application and spiking with primary isolates representing different clades with virus diluted in seminal plasma.

Although the precise mechanisms by which HIV and other STI pathogens gain access to their target cells has not been fully elucidated, the epithelium provides an important first line of defense. Virus must overcome the protective multilayered vaginal epithelium and the tight junctions between epithelial cells, which provide a relatively impervious barrier to HIV-1 target cells, which include dendritic cells, T cells and macrophages. The epithelial barrier and tight junctions may be disrupted in the setting of other STI or following exposure to hyperosmolar vaginal products or microbicides (14). The importance of this barrier is highlighted by recent *in vitro* and murine studies from our lab.

We have developed a dual-chamber culture system to evaluate the impact of microbicides on the tight junctions and on the subsequent ability of HIV to traverse this barrier and infect target T cells cultured in the lower chamber. In our initial studies, we evaluated two drugs that have completed clinical trial testing and were associated with an increase risk of HIV acquisition (N-9 and CS) and two drugs that are currently in Phase II/III clinical studies, tenofovir (phosphonylmethoxy propyl adenine monohydrate, or PMPA) and PRO 2000. The results obtained indicate that this dual chamber model would have predicted the outcome of both the N-9 and CS trials. We observed that N-9 (0.01%) and CS (100 μg/l) disrupted tight junctions as demonstrated by a drop in transepithelial resistance (TER), which was accompanied by disruption and down-regulation of several junctional proteins as measured by quantitative real-time polymerase chain reaction (qRT-PCR). The disruption of the tight junctions persisted for several days, even after CS was removed from the cultures, and resulted in enhanced migration of HIV particles across the barrier with productive infection of T cells cultured in the lower chamber. The disruption was confirmed by confocal microscopy studies. Tenofovir (PMPA) had no deleterious effects on the epithelial integrity and the epithelium remained relatively impervious to HIV migration. At a comparable concentration (100 μg/ml), PRO 2000 also had little or no effect on the integrity of tight junctions, although increasing the concentration to 1 mg/ml did result in transient reduction in the TER. Both CS and PRO 2000 triggered a modest increase in inflammatory mediators with activation of NF-kappaB pathways, but the inflammatory response was not of sufficient magnitude to increase HIV p24 expression in persistently infected U1 cells.

We have translated these studies to a murine model and have shown that repeated vaginal applications of 3.5% N-9, but not 1% tenofovir gel nor 0.5% PRO 2000 gel, results in disruption of the mouse lower genital tract with down-modulation of junctional proteins and results in enhanced susceptibility to HSV. Together, these findings provide a biological explanation for the results obtained in the Phase III N-9 study as well as the unanticipated clinical trial result indicating an increased risk of HIV acquisition with CS. Further, our expanded preclinical models suggest that 1% tenofovir gel and 0.5% PRO 2000, which are both currently in large-scale clinical trials, will prove safe with respect to their effects on the epithelial barrier. However, frequent exposure to higher concentrations of PRO 2000 may result in a more sustained impact on epithelial integrity. The dual chamber and murine models require validation in human trials in which the epithelium can be evaluated via biopsy pre- and post-application of candidate agents.

Safety assessment of candidate microbicides and formulations typically includes an evaluation of the effects on lactobacilli *in vitro* and on prevalence and incidence of bacterial vaginosis (BV), *Candida* or *Trichomonas* in Phase I/II clinical studies. A few studies have also included semiquantitative cultures of select bacteria. For example, in a Phase I safety study of CS, semiquantitative cultures demonstrated no alterations in H_2_O_2_-negative lactobacilli, *Gardnerella vaginalis*, *Enterococcus*, anaerobic gram-negative rods, *Mycoplasma hominis* or group B streptococci among subjects who applied CS and K-Y Jelly in a 14-day trial (15). However, women in both treatment groups had decreased levels of H_2_O_2_-positive lactobacilli compared to baseline and notably, participants in the CS group had statistically significantly increased odds for *Escherichia coli* and *Staphyloccocus aureus* compared to those in the K-Y group. Whether the increase in *E. coli* and *Staph aureus* reflected a loss in the endogenous antimicrobial activity and/or a broader shift in the genital tract microbial community and whether this change contributed to the enhanced HIV acquisition observed in the Phase III trial can only be speculated. However, these findings underscore the need to incorporate assays that quantitatively evaluate the impact of vaginal products and microbicides on this microenvironment *in vivo*. Quantitative cultures are labor intensive and may not identify infrequent or minority bacterial species or those that are difficult to culture. Taxon-directed 16S rRNA gene quantitative PCR assays have been developed for the detection of key vaginal bacteria. Using molecular approaches, Dr. David Fredricks and his colleagues have identified several novel bacterial species in subjects with BV (16, 17). These methods permit the detection of microorganisms that are not easily cultured and thus provide a more comprehensive description of the vaginal microflora. In the proposed clinical study, we will employ this approach to better define the vaginal flora before and after intravaginal application of Acidform lubricant or HEC gel.

**2.2 Acidform Lubricant and Hydroxyethylcellulose Gel Prior Research**

Preclinical studies provide evidence of the contraceptive and antimicrobial activity of Acidform gel (7, 8). Comparative studies demonstrate Acidform gel’s greater spermicidal activity compared to Aci-jel® (Ortho McNeil Pharmaceuticals Raritan, NJ) and BufferGel™ (ReProtect LLC, Baltimore, MD), as expected, due to its higher acid-buffer capacity (7, 8). In titration studies, about twice as much sodium hydroxide is required to bring the pH of Acidform gel up to 5.0 as compared to BufferGel and about four times as much as compared to Aci-jel (7, 8). In semen dilution studies, a 3-fold excess of semen is required to bring the pH of Acidform gel up to 5.0, whereas only an equal amount of semen is necessary for BufferGel and even less for Aci-jel (8).

In addition to maintaining an acidic pH after semen deposition, Acidform gel’s bioadhesive and viscosity-retaining characteristics make it an excellent vaginal delivery system. Its bioadhesive qualities ensure the formation of a protective layer and prolonged retention in the vagina. The gel maintains good viscosity upon dilution, suggesting that deposition of semen in the vagina will cause neither a decrease in gel thickness nor excessive leakage (7, 8).

Acidform gel has a broad spectrum of antimicrobial activity *in vitro*, including activity against herpes simplex virus-type 2 (HSV-2), C*hlamydia trachomatis*, *Neisseria gonorrhoeae* and BV-causing microbes without having a detrimental effect on lactobacillus (7, 10). One GLP animal safety study, the 10-day rabbit vaginal irritation study, was conducted with Acidform gel (7). The degree of vaginal irritation for Acidform gel was mild and within the acceptable range.

A Phase I blinded, single center, randomized clinical study evaluating the capability of Acidform gel compared to a Brazilian commercially available product (2% N-9) to immobilize sperm in the postcoital test (PCT) was performed (18). In this study, twenty subjects completed a baseline cycle and 3 treatment cycles: 1) Acidform gel inserted 0-2 hours before intercourse; 2) Acidform gel inserted 8-10 hours prior to intercourse; and 3) 2% N-9 inserted 0-2 hours prior to intercourse. The aims of the study were to assess the effectiveness of Acidform gel compared to the control in preventing sperm from penetrating mid-cycle cervical mucus and to collect information on symptoms and signs of irritation of the female genitalia. Endpoints were the number of progressively motile sperm in the cervical mucus, findings on colposcopy, presence of inflammatory markers in the vaginal lavage, and changes in vaginal microflora and pH. There were statistically significant differences (p<0.01) between the number of progressively motile sperm in the three test group PCT results compared to the baseline cycles and no statistical difference between Acidform gel inserted 0-2 hours before intercourse and N-9. While both products significantly reduced sperm levels compared to baseline, Acidform gel inserted 8-10 hours before intercourse was not as effective as N-9 at inhibiting progressively motile sperm (p=0.01).

One participant out of 20 women who used Acidform gel (0-2h) reported 1 minor symptom of vaginal burning. Four out of 20 women who used Acidform gel (8-10h) reported 9 minor symptoms including vaginal burning (2), unpleasant odor (2), pain (2), vaginal dryness (1), difficulty reaching orgasm (1), and clitoral irritation (1). In comparison, 5 out of the 20 women using N-9 reported 7 minor symptoms including vaginal burning (2), abdominal pain (1), pruritis (1), vaginal dryness (1), and reduced sensitivity/pleasure (2). On colposcopic examination, 4 findings of erythema and no findings that disrupted the epithelium were observed in two treatment cycles of Acidform gel.

Three male participants exposed to Acidform gel in two treatment cycles experienced 4 minor symptoms including genital burning (1), reduced sensitivity (2), and erectile dysfunction (1). In comparison, 6 male participants exposed to N-9 in one treatment cycle experienced 6 minor symptoms including genital burning (2), reduced sensitivity/pleasure (3), and an uncomfortable sensation (1).

Acidform gel appeared to be at least as safe for penile exposure as the marketed lubricant K-Y Jelly in a double blinded, Phase I clinical safety study conducted at Advances in Health, Inc., in Houston, Texas (19). In this study, 36 sexually abstinent male volunteers were randomly assigned in a 2:1 ratio to 2 ml of Acidform gel or K-Y Jelly applied to the penis daily for 7 consecutive days. There were 2 complaints in 2 men, which were tingling and penile dryness, in the group of 24 men assigned to Acidform gel and there were 9 complaints in 5 men, which were itching, tingling and burning, in the 12 men assigned to K-Y Jelly. Two of 24 (8.3%) male volunteers in the Acidform gel group had possibly product-related findings on genital examination, which were a 1 mm ulcer and a cluster of vesicles. One of 12 (8.3%) of male volunteers in the K-Y Jelly group had a possibly product-related finding of erythema on genital examination.

Nonclinical studies conducted to date indicate that HEC gel is quite safe (20). Exposure of a human vaginal epithelial cell line to HEC gel did not result in cytotoxicity or induction of the proinflammatory cytokine IL-1α. The gel also had minimal effect on sperm motility and displayed no or negligible anti-HIV activity *in vivo*. Intravaginal application of HEC gel did not increase susceptibility to vaginal HSV-2 infection in mice, and daily intravaginal application for 10 days was not irritating to the vagina in rabbits.

**2.3 Potential Risks and Benefits**

## Benefits

There may be no direct benefits to participants in this study. However, participants and others may benefit in the future from information learned from this study. Specifically, information learned in this study may lead to the incorporation of Acidform as a vehicle for formulating safe and effective candidate microbicides that prevent sexual transmission of HIV and other STI.

Participants will receive HIV counseling and testing as part of the study screening process as well as pelvic exams, including a Pap test. Participants also will be screened for STI, vaginal and urinary tract infections and provided treatment if applicable.

**Risks to Human Subjects**

Genital symptoms including vaginal burning, unpleasant odor, pain, vaginal dryness, difficulty reaching orgasm and clitoral irritation were reported in a few women who used Acidform in a postcoital study.

In a Phase I clinical trial to assess the safety of HEC gel, no serious or unexpected adverse events were reported following twice-daily intravaginal application for two weeks in 14 healthy, sexually abstinent women (21). Two out of 14 women (14%) had at least one symptom of genital irritation. All of the genital symptoms were considered to be of mild severity and included genital pain, burning, soreness or pelvic pain. In a pilot study in preparation for use as a placebo in Phase III microbicide trials there were no serious adverse events reported with use of HEC gel.

Other risks include the possibility of pain, bleeding and infection following biopsy of the cervix.

##

## 3.0 OBJECTIVES

## 3.1 Primary Objective

The primary objective of the study is to compare the safety of Acidform lubricant to HEC gel by assessing the genital tract mucosal response to 14 days of twice daily application by measuring endogenous antimicrobial activity and levels of mediators of host defense, including defensins, cytokines, and chemokines in CVL.

**3.2 Secondary Objective**

To assess the extent and duration of buffering by measuring vaginal pH prior to and 2 hours after the first application of Acidform or HEC.

**3.3 Exploratory Objectives**

To determine whether intravaginal application of Acidform lubricant or HEC results in changes in epithelial integrity by collecting cervical biopsies.

To evaluate changes in vaginal flora following repeated intravaginal application of Acidform lubricant or HEC.

To examine the antiviral activity in CVL collected 2 hours following a single dose application of Acidform lubricant or HEC gel using a spiking strategy.

**4.0 STUDY DESIGN**

This is an open-label randomized comparative study of Acidform lubricant and HEC gel to be conducted among 36 women at 2 clinical sites, the Albert Einstein College of Medicine (AECOM) and Montefiore Medical Center (MMC). The female participants will have a normal gynecological history and be at low risk for HIV or other STI. Participants will be randomized to apply Acidform or HEC twice daily for 14 consecutive days between menses. Instead Healthcare, LLC (La Jolla, CA) will provide Acidform lubricant and HEC gel. The randomization will be 1:1 (i.e., 18 in each treatment arm). Participation in this study will last approximately two months.

Prior to screening, potential participants will be informed of the need to abstain from insertion of anything into their vagina from 48 hours prior to the enrollment visit until completion of the study. Women unwilling or unable to assure abstinence will not be enrolled. Participants must agree to abstain from vaginal or anal intercourse or insertion of sexual toys. They must also agree not to douche or use any vaginal product other than the investigational product including lubricants, feminine hygiene products, and vaginal drying agents for 48 hours before enrollment and for the duration of the study (21 days).

At the screening visit, eligible volunteers will come to the General Clinical Research Center (GCRC) at the AECOM or MMC. After informed consent is obtained for the study and for HIV testing, a medical, gynecological, and sexual history will be obtained. Urine will be collected for microscopy, culture, and a pregnancy test. If the pregnancy test is negative, a pelvic examination will be performed including a speculum exam with visual inspection, pH measurement, wet mount, and collection of a baseline cervicovaginal lavage (CVL) sample. CVL is a wash of the cervicovaginal area using 10 ml of normal saline. A 10 ml syringe filled with saline is aimed directly at the cervical os and posterior vaginal wall. The pooled fluid is aspirated and the volume is collected. A Pap test will be performed if documentation of a negative result within the last year is not available. A cervical swab for nucleic acid amplification testing for *N. gonorrhoeae* and *C. trachomatis* will be done. One biopsy will be obtained from the ectocervix near the external os. Dr. Mark Einstein or Dr. Rodney Wright will collect the cervical biopsy colposcopically. If there is evidence of a clinically detectable genital abnormality (i.e., ulcer, lesion or positive wet mount) or urinary tract infection, appropriate treatment and follow-up will be provided and the volunteer will be ineligible to continue in the study at this time. If a lesion or ulcer is present at this or any visit, a herpes culture will also be performed. Blood will be collected for a pregnancy test, HIV EIA, RPR, HSV serologies (type common and type-specific; Focus Technologies, Herpes Select) (See Appendix I). Subjects with no contraindications to study participation will be presumed eligible for enrollment.

On the day of Enrollment (Day 0) (2-6 days after the next menses, within 30 days of screening), participants will return to the GCRC. Test results from screening will be reviewed. Urine will be collected for a repeat pregnancy test. If the pregnancy test remains negative, a speculum exam will be performed and the vagina and cervix will be visually inspected. A pH sample from the vaginal wall and a gentle swab for wet mount will be obtained. A vaginal swab will be collected for assessment of vaginal flora and a cytobrush will be obtained to measure immune cell populations. If there is evidence of a clinically detectable genital abnormality (i.e., ulcer, lesion or positive wet mount) appropriate treatment and follow-up will be provided and the participant will be ineligible to participate further at this time. If there is evidence of recent intercourse (sperm visualized on wet mount or positive p30 test), the participant will be ineligible to continue in the study. If the patient is eligible, participants will be randomized to receive Acidform lubricant or HEC gel. The participant will insert one dose of study gel intravaginally under the guidance of the study clinician. A repeat pelvic exam with visual inspection, measurement of vaginal pH and collection of CVL will be performed 2 hours after application of Acidform or HEC gel. Provided that the first dose is well tolerated, the participants will insert subsequent applications twice daily for 13 consecutive days (preferably in the morning and at bedtime) (see Appendix III). Subjects will be instructed and frequently reminded to apply one dose in the morning and a second dose at bedtime. Study gels will be provided in single-use tubes with separate vaginal applicators and the participant will be provided with a study diary to record usage, symptoms and reasons for non-adherence.

Every effort will be made to bring participants back after 7 and 14 doses of Acidform or HEC. Visits will be scheduled in the GCRC on Day 7 (range, days 5-9) and Day 14 (range, days 12-15). For study visits conducted on Day 7 and 14, subjects will be instructed to apply the morning dose after the study visit is completed. There will be a Final Visit scheduled on Day 21 (range, days 16-21), prior to the anticipated onset of menstruation. A pelvic exam will be performed and vaginal pH will be measured at each visit. CVL samples will be obtained at the Screening Visit, Enrollment Visit (2 hours after the application of Acidform or HEC), and on Days 7, 14, and 21. A cytobrush for measurement of immune cell populations and a vaginal swab for assessment of vaginal flora will be collected at Enrollment (prior to application of Acidform or HEC), Day 14 and Day 21. Dr. Mark Einstein or Dr. Rodney Wright will collect one cervical biopsy colposcopically at the Day 14 visit for assessment of epithelial integrity. Participants will be instructed to document symptoms of vulvar or vaginal soreness or pruritus, vaginal bleeding, dysuria, or acts of vaginal penetration in a study diary, which will be provided. Participants will be asked to record information on the number of times they administer study gel, time of each insertion, emergence/resolution of specific symptoms and reasons for non-adherence. Diaries will be reviewed at each visit. Participants will bring used and unused supplies with them to each visit for counting and disposal. Adverse events will be recorded as described in Section 8.0. Subjects will receive $25 at the Screening visit. An additional $25 will be given at Screening if a biopsy is performed. Fifty dollars will be provided at the Enrollment, Day 7, Day 14 and Day 21 study visits. An additional $50 will be given for completion of a biopsy at the Day 14 study visit. A total of $300 will be provided for completion of all study visits as compensation for their time and travel expenses.

**4.1 Laboratory Methods**

The vaginal pH will be measured using an electronic ISFET pH meter. A stainless steel sensor pH probe will allow us to measure the pH in different locations in the vagina. There is less variability with the probe compared to pH paper, which has been used in most of the previous studies. We will measure pH at Screening, Enrollment (prior to and 2 hours after the first application of Acidform or HEC), Day 7, Day 14 and Day 21.

CVL will be immediately transported on ice to the investigators’ laboratories and centrifuged for 20 minutes at 1000 g at 4°C, aliquoted and stored at -80°C. CVL will be collected using 10 ml of normal saline. We have demonstrated that Acidform gel retains its low pH (~ 3.5) when mixed 1:10 with normal saline. Thus, collection of CVL in normal saline should not interfere with the buffering effects of Acidform. Cytobrush specimens will be transported on ice to the lab. The cells will be gently dislodged, centrifuged and immediately frozen in fetal bovine serum with 10% dimethyl sulfoxide. The cells will be thawed at a later date, counted, washed, respun and stained using a panel of differentially conjugated mAbs against various human leukocyte membrane antigens and analyzed by fluorescent activated cell sorter (FACS). Cell preparations will be labeled in parallel with appropriate isotype control Abs (Becton-Dickinson).

Cytokines and chemokines in CVL supernatants will be quantified using a multiplex Luminex assay (Millipore), which allows for the quantitative measurement of multiple cytokines or chemokines using a small sample size. The following mediators will be measured by Luminex: IL-1α, IL-1β, IL-2, IL-6, IL-8, IFN-αβ, IFN-γ, and RANTES. IgG, IgA, SLPI, IL-1ra, lactoferrin, lysozyme and HNP1-3 also will be measured using commercial ELISA kits (R & D Systems, Minneapolis, MN). These mediators were selected because of their potential to influence mucosal immunity. The total protein concentration will be measured and the results will be presented per mg of protein in CVL as well as per ml of CVL to control for any differences in CVL collection. To determine whether Acidform or HEC interferes with the detection of any of the mediators, standards will be diluted in saline or saline containing Acidform or HEC gel (2 ml of Acidform or HEC added to 10 ml of normal saline) and results will be compared.

CVL will be assayed for endogenous antimicrobial activity, focusing on the ability of CVL to inhibit HSV and *E. coli*. HSV and *E. coli* were selected based on prior studies from our laboratory. For the endogenous anti-herpes activity, human cervical epithelial cells (CaSki) will be treated with each CVL sample or control buffer (normal saline containing 2 mg/ml of albumin to control for total protein concentration) and then challenged with serial dilutions of HSV-2(G) in duplicate. The ability of the CVL to inhibit viral infection will be determined by comparing the viral titer (pfu/well) in the presence of each CVL sample to that in the presence of control buffer and will be determined by counting plaques 48 h post-infection. The effects of CVL pre- and post-application of Acidform or HEC gel on the growth of *Escherichia coli* ML-35 will also be examined. Serial dilutions of the bacteria will be incubated with an equivalent volume of CVL or control fluid at 37°C for 2 h before plating on agar plates. Colonies will be counted using ImageQuant TL v2005 after an overnight incubation at 37°C and relative number of colonies will be compared. It should be noted that the CVL is collected ~10-12 hours post-application of Acidform or HEC and thus the antimicrobial activity should primarily reflect changes in innate immunity, rather than the direct effects of the gel. However, a contribution from residual gel cannot be excluded.

To assess the activity of the gels themselves, we will compare the antiviral activity of Acidform or HEC in CVL obtained 2 hours after the first application using a spiking strategy. The experimental design is based on our prior studies with 0.5% PRO 2000 gel (12). Serial dilutions of each CVL supernatant will be spiked with dilutions of HIV-1 or HSV-2 (diluted in saline or seminal plasma), assayed for pH and then inoculated onto susceptible cells. For HIV, we will use TZM-bl cells expressing CCR5 or CXCR4 and CD4 and infect with the appropriate R5 or X4 viruses. For HSV-2 studies, we will use a human cervical epithelial cell line. The viral titer will be determined by counting plaques 48 h post-infection after immunostaining by black plaque assay. Seminal plasma collected from low risk males who participated in a previous unrelated study will be used for these experiments. These studies will also be expanded to include primary isolates representative of different clades and primary cell cultures.

To assess epithelial integrity, cervical biopsy specimens will be split into two sections. One half will be used for confocal studies and the other half for real-time PCR studies. The biopsy tissue will be stained with EZ-Link sulfosuccinimidobiotin reagent, which reacts with primary amines on cell-surface proteins. Following membrane labeling, the tissue will be fixed with paraformaldehyde. To visualize junctional proteins, the tissue will be permeabilized with 1% Triton X after fixation, incubated with the appropriate primary Abs and subsequently with an Alexa Fluor 488-conjugated anti-mouse secondary Ab. Nuclei will be detected by staining with DAPI nucleic acid stain. Imaging studies will be conducted in the Microscopy Core Facility. The other half of the biopsy sample will be homogenized and total RNA extracted and levels of mRNA transcripts determined by quantitative real-time PCR. Expression levels of cellular architectural proteins including E-cadherin, ZO-1, nectin, and others will be determined using established primer and probe sequences with the inclusion of an appropriate housekeeping gene as an endogenous control.

We will also compare differences in vaginal flora pre- and post-application of Acidform lubricant and HEC gel. Vaginal swabs will be collected at Enrollment, Day 14 and Day 21, stored at -80°, batched and transported to Dr. David Fredrick’s laboratory at the Fred Hutchinson Cancer Research Center for analysis. Briefly, the vaginal swabs will be diluted in saline, vortex mixed, and centrifuged at 14,000 g for 10 minutes and DNA will be extracted from the pellet using the Ultra Clean Soil DNA Kit (MoBio). Taxon-directed real time quantitative 16S rRNA gene PCR assays will be utilized to detect and quantify *Lactobacillus crispatus*, *Gardnerella vaginalis*, *E. coli*, and BVAB1, BVAB2 and BVAB3 using a modification of previously described assays that have been validated in the lab (16, 17). These assays employ a fluorescent TaqMan probe to monitor production of the intended bacterial 16S rDNA product with each PCR cycle, allowing construction of a standard curve based on known quantities of bacterial 16S rDNA, and the calculation of bacterial concentrations in vaginal samples. Organisms were chosen to target bacteria commonly found in healthy women (*L. crispatus*, *G. vaginalis*, *E. coli*). In addition, we selected organisms specific to BV (BVAB1-3) given the possibility of encountering asymptomatic BV or altered flora in the proposed clinical study.

**5.0 STUDY POPULATION**

Thirty-six healthy women will be enrolled in this study. Participants will be selected for this study and recruited according to the criteria below. Conditions for withdrawal from the study are described in Section 12.

Subjects will be recruited from the catchment area of the MMC, which is centered around 2 hospital campuses, the Montefiore Hospital and the Weiler Hospital. There is a diverse ethnic distribution consisting of 48% Hispanics, 36% Blacks and 7% Whites. The diversity of the population in our catchment areas permits the recruitment of patients from most of the major ethnic groups including Asians and Pacific Islanders, Blacks, Hispanics from Mexico, Puerto Rico, Cuba and Central and South America and Caucasians.

The study will be conducted at the General Clinical Research Center (GCRC) at the AECOM and MMC. Providers from the Internal Medicine, Adolescent and Gynecology Clinics at MMC will refer participants. IRB-approved advertisements will be posted to recruit self-referrals. Potential subjects will contact the study staff to discuss the details of the study, including inclusion criteria, and to schedule screening appointments.

# 5.1 Participant Inclusion Criteria

# Women who meet all of the following criteria (by self-report, unless otherwise specified) are eligible for inclusion in this study:

- Age between 18 and 50.
- Able and willing to provide written informed consent to participate in the study.
- Normal menstrual history with regular cycles with a minimum of 21 days between menses.
- Low risk for HIV/STI infection. Low risk is defined as

1. Not having had an STI in the last 6 months.
2. Not having used non-therapeutic intravenous drugs in the last year.
3. Being either sexually abstinent or being in a stable, mutually monogamous relationship with a partner who is not known to be HIV positive.

- Agree to the insertion of Acidform lubricant or HEC gel.
- Agree to abstain from vaginal and anal intercourse and to not use vaginal products for 48 hours prior to enrollment and for the duration of the study (through the Final Visit).

# 5.2 Exclusion Criteria

Women who meet any of the following criteria (by self-report, unless otherwise specified, at screening, enrollment, or at anytime during participation, unless otherwise specified) will be excluded from this study:

- Are pregnant (based on a serum or urine pregnancy test) or less than 6 months post-partum.
- Are currently breastfeeding.
- Are menopausal.
- Are currently using hormonal contraception or have used hormonal contraception in the previous 2 months.
- Are currently menstruating (at screening or enrollment visits – may be eligible for deferment).
- Are currently participating in a research study of other vaginal products.
- Have a positive HIV test at screening.
- Have a urinary tract infection at screening (may be eligible for re-screening after treatment).
- Have a positive chlamydia, gonorrhea, or trichomonas result at screening.
- Have an abnormal Pap test (if documentation of a negative result within the past year is not available).
- Have a clinically detectable genital abnormality (i.e., vulvar, vaginal, cervical and/or perianal ulcer and/or lesion or an abnormal wet mount).
- Have a history of intermenstrual bleeding in the 3 months prior to enrollment.
- Have had gynecological surgery or received treatment for syphilis, genital herpes, chlamydia, gonorrhea, trichomonas, or genital warts in the last 6 months.
- Have received treatment for candida, bacterial vaginosis, or urinary tract infection in the prior month.
- Have douched or used any vaginal products**,** including lubricants, feminine hygiene products, vaginal drying agents and sex toys during the 48 hours prior to enrollment.
- Have had vaginal or anal intercourse during the 48 hours prior to enrollment.
- Oral or intravenous antibiotics in the 7 days prior to enrollment.

**6.0 STUDY PRODUCT/INTERVENTION(S)**

**6.1 Regimen**

Study staff will review the administration of Acidform lubricant or HEC gel with each participant at her Enrollment Visit. At the Enrollment Visit, the participant will insert a 5 gram (equal to 5 ml) dose of Acidform lubricant or HEC gel vaginally to a depth of approximately 6 cm under the guidance of the study clinician. The volunteers will insert subsequent applications twice daily for a dosing total of 14 days. Participants will be instructed on proper insertion technique and storage requirements (see Appendix III).

**6.2 Study Product Formulation and Preparation**

**Acidform**

Acidform is an acid buffered gel (pH = 3.5) containing three buffering agents (citric acid, potassium bi-titrate, and lactic acid). It is dense, viscous, off-white, and uniform with an acidic flavor (8). The acid-buffering properties of Acidform gel maintain the acidic vaginal milieu (pH = 3.5-4.5) even in the presence of semen, making the gel spermicidal. Its antimicrobial action towards herpes and chlamydia have been established through animal studies using Acidform gel without N-9 (7).

Product should be stored at controlled room temperature until required for administration. Controlled room temperature is defined as 25 degrees Celsius (°C)/ 77 degrees Fahrenheit (°F). Excursions are permitted to 15°C to 30°C (59°F to 86°F).

**HEC**

The placebo for this study is hydroxyethylcellulose (HEC) gel. HEC gel is a water-based gel formulation designed for use as an inert comparator in clinical trials of candidate vaginal microbicides for the prevention of STI, including HIV infection. HEC gel was designed to neither increase nor decrease the risk of HIV infection, but to sufficiently match the physical properties of candidate active products so as to control for behavioral confounders in clinical trials. The formulation was developed by Dr. Thomas Moench (ReProtect, Inc.) and will be provided by Instead Healthcare, LLC.

HEC gel is similar in appearance and physical properties as the marketed over-the-counter vaginal lubricant K-Y Jelly (McNeil-PPC, Inc.). All ingredients used in the manufacture of HEC gel are compendial (United States Pharmacopeia/National Formulary) and are generally recognized as safe for use in topical pharmaceutical preparations. Hydroxyethylcellulose is used as a gel thickener. HEC gel was made isotonic (with sodium chloride) to reduce the risk of epithelial damage caused by cell swelling or dehydration. The formulation was also made slightly acidic (pH 4.4) to avoid disrupting the normal vaginal pH. Buffering capacity, however, is minimal. Sorbic acid was selected as a preservative based on its safety profile and lack of activity against HIV *in vitro*.

Placebo gel must be stored at controlled room temperature at all times. Controlled room temperature is defined as 25 degrees Celsius (°C)/ 77 degrees Fahrenheit (°F). Excursions are permitted to 15°C to 30°C (59°F to 86°F).

**6.3 Study Product Supply, Packaging and Accountability**

Instead Healthcare, LLC will provide Acidform lubricant, HEC gel and applicators for this study. Acidform lubricant and HEC placebo gel will be prepared in compliance with Good Manufacturing Practice (GMP) regulations for Instead Healthcare, LLC by Swiss-American Products, Inc, Carrollton, TX.

Study gel (Acidform or HEC) will be packaged in cartons containing 30 pre-filled plastic applicators. The AECOM Research Pharmacist will randomly allocate Acidform or HEC gel in a 1:1 fashion (i.e., 18 subjects per arm).

The pharmacist will maintain an accurate inventory and accountability record of study products received and subsequently dispensed. After the study is completed (or otherwise terminated), all unused study product will be shipped to Instead Healthcare, LLC for destruction. Participants will be dispensed thirty pre-filled applicators of study product at the Enrollment Visit. All product, used and unused, will be returned by the participants to AECOM or MMC. The subjects will be counseled that they are to apply 28 doses of drug or placebo. They will be instructed that they will be given 2 extra doses of drug or placebo to use only if a dose is dropped or lost.

6.4 Assessment of Participant Adherence with Study Product/Intervention(s)

The first application of investigational product will be done at the Enrollment Visit by the participant under the guidance of the study clinician. Participants will be instructed to apply the assigned investigational product at bedtime and twice daily for 13 subsequent days (see Appendix III). For study visits conducted on Day 7 and 14, subjects will be instructed to apply the morning dose after the study visit is completed.

Participants will be asked to record information on the number of times they administer study gel, time of each insertion, and emergence/resolution of specific symptoms in a study diary. Diaries will be reviewed at each visit.

Non-adherence will be defined as three or more days (out of 14) of missed product use. Participants who miss less than three days will (if practical according to their expected date of menses onset) be asked to apply product for additional days to attempt to achieve a full 14 days of product exposure. An additional participant will be recruited for each non-adherent participant. Participants who are non-adherent with the study protocol will be asked their reasons for non-adherence and this information will be recorded on the applicable study forms. Study procedures for Day 7, Day 14, and the Final Visit will be re-scheduled as necessary. Participants will be instructed to call the study staff in the event of missed doses to re-schedule study visits.

Participants will be instructed to abstain from sexual relations throughout the course of the study. To assess whether intercourse occurred, a wet mount will be performed to look for sperm and a vaginal swab will be collected and tested for the presence of semen using the ABAcard p30 test. The test is an antibody immunoassay that detects p30, a glycoprotein produced by the prostate, which is present in human semen. p30 is detectable in the vaginal tract up to a maximum of 2 days. If a participant is found to have engaged in anal or vaginal sexual relations or experienced vaginal penetration with anything other than the study gel applicator throughout the study, this information will be recorded on applicable case report forms. Participants who are non-adherent to sexual abstinence on more than one occasion will discontinue product use. An additional participant will be recruited for each non-adherent participant.

A participant with a clinical toxicity outcome (see Appendix IV) who is discontinued from further product use will be considered to have reached a study endpoint and will not be replaced. Standard clinical care will be provided and followed to resolution.

## 6.5 Concomitant Medications

Enrolled participants may continue use of all concomitant medications except vaginal products (lubricants, feminine hygiene products, and vaginal drying agents) during this study. All concomitant medications (including complementary and alternative medicines, vitamins, etc.) will be recorded on applicable study case report forms. Medications used for the treatment of AEs that occur during study participation also will be recorded on applicable case report forms.

**7.0 STUDY PROCEDURES/EVALUATIONS**

**7.1 Clinical Evaluations and Procedures**

See Appendix I for a complete Schedule of Procedures and Evaluations by study visit.

Interested volunteers will contact the study staff to discuss study details, including inclusion criteria, prior to the screening visit. Potential participants will be screened for presumptive eligibility for the study according to the procedures described below. All procedures will be completed in a step-wise manner for potential participants who meet the study eligibility criteria. For participants who do not meet the eligibility criteria, screening will be discontinued when ineligibility is determined. For potential participants who are found to be presumptively eligible at this visit, final eligibility will be confirmed at an Enrollment Visit scheduled to take place 2-6 days following the next menses within 30 days of the Screening Visit.

## Screening Visit

## Clinical Procedures

- Obtain informed consent.
- Assign Participant ID.
- Collect demographic and locator information.
- Collect medical history and concomitant medication information.
- Collect blood for pregnancy test, HIV EIA, RPR, herpes serology
- Collect urine for HCG pregnancy test; provide participant with result.
- Collect urine for microscopy and culture.
- Perform pelvic examination including:
- Speculum examination of vagina and cervix,
- Collection of pH sample from the vaginal wall, anterior and posterior fornices,
- Swabs from anterior or lateral fornix for wet mount (two samples of discharge on slide for saline prep, potassium hydroxide prep and whiff test), & p30 test for detection of semen (One-Step ABA card PSA, Abacus Diagnostics, West Hills, CA).
- Collection of baseline CVL,
- Pap test (if documentation of a negative result within the past year is not available),
- Cervical swab for *N. gonorrhoeae* and *C. trachomatis*,
- If ulcerative lesions are noted, collect swab for herpes simplex virus culture,
- Colposcopically directed cervical biopsy.
  - If participant determined to be ineligible, end visit and complete all required data collection forms. If participant remains presumptively eligible, schedule Enrollment Visit to occur 2-6 days following menses within 30 days after the Screening Visit.
- Instruct participant not to use vaginal products and to abstain from vaginal and anal intercourse for 48 hours prior to the Enrollment Visit.
- Complete all required data collection forms.

### Laboratory Procedures

- Record results of serum and urine pregnancy tests.
- Record results of urine microscopy and culture.
- Record vaginal pH measurements.
- Examine wet mount by direct microscopy to detect *T. vaginalis*, *C. albicans* infections, and clue cells for BV. Perform whiff test.
- Conduct p30 test for detection of semen.
- Conduct tests for *N. gonorrhoeae* and *C. trachomatis*.
- Read and record Pap test results.
- Conduct culture of swab for HSV, if clinically indicated.
- Conduct serology for RPR with confirmation by FTA-ABS or TPHA for *T. pallidum.*
- Conduct type-specific herpes serology.
- Conduct EIA test for HIV.
- Process, spin, measure volume, aliquot and freeze CVL supernatants, which will be used for assessment of endogenous antimicrobial activity, cytokines, chemokines and mediators of mucosal immunity.
- Process cervical biopsy tissue for assessment of epithelial integrity by microscopy and qRT-PCR.

Enrollment Visit (Day 0)

Participants who are found to be presumptively eligible at their Screening Visits will complete Enrollment Visits within 30 days after the Screening Visit. In order to ensure a minimum of 21 days without menstrual bleeding, participants will be scheduled for an Enrollment Visit 2-6 days after the next menstrual period ends. Participants who do not complete an Enrollment Visit within 30 days of screening must repeat the screening process in order to participate. All participants will receive their screening test results at their Enrollment Visits. For those whose test results meet the study eligibility criteria, the procedures below will be undertaken in a step-wise manner to confirm eligibility. As was the case at the Screening Visit, the procedures will be discontinued if ineligibility is determined at this visit.

## Clinical Procedures

- Update locator information.
- Review interim medical history and concomitant medication information.
- Provide participant with screening test results.
- Collect urine for HCG pregnancy test; provide participant with result.
- Perform pelvic examination including:
- Speculum examination of vagina and cervix,
- Collect pH sample from the vaginal wall, anterior and posterior fornices,
- Gently swab anterior or lateral fornix for wet mount (two samples of discharge on slide for saline prep, potassium hydroxide prep, whiff test, and microscopic inspection for sperm) and p30 test for detection of semen,
- Collect vaginal swab for evaluation of vaginal flora,
- Collect cytobrush.
- If patient determined to be ineligible, end visit and complete all required data collection forms. If participant remains eligible, randomize/assign Enrollment ID and continue.
- Distribute study supplies, including thirty (30) single-application tubes of Acidform gel or placebo gel and 30 single-use applicators, study diary, application and storage instruction sheet (Appendix III) and panty liners.
- Instruct participants to bring study diary and used and unused tubes of gel to all follow-up visits.
- Provide information regarding product administration.
- Provide guidance for the administration of the first dose of study gel by the participant.
- Measure vaginal pH and collect CVL 2 hours post-application of Acidform or HEC.
- Instruct participant on anticipated amount, consistency and color of discharge due to gel expulsion.
- Instruct participant on proper gel insertion techniques and supply storage.
- Instruct participant to apply a dose at bedtime and then twice daily starting the next morning.
- Schedule Day 7 Study Visit.
- Instruct participant to not apply a dose on the morning of the Day 7 Visit. The dose will be applied after the study visit to avoid the effects of residual drug on study samples.
- Complete all required data collection forms and record any AE information.

## Laboratory Procedures

- Record results of urine pregnancy test.
- Record vaginal pH measurements prior to application of Acidform or HEC.
- Examine wet mount by direct microscopy to detect *T. vaginalis*, *C. albicans* infections, clue cells for BV, and/or sperm and record results. Perform whiff test and record results.
- Conduct p30 test for detection of semen.
- Store vaginal swabs for vaginal flora assessment. Swabs will be batched and sent to Dr. Fredricks at the University of Washington at the end of the study.
- Record vaginal pH 2 hours post-application of Acidform or HEC.
- Process, spin, measure volume, aliquot and freeze CVL supernatants for spiking studies.
- Process cytobrush for evaluation of cell populations by FACS.

## Follow-up Visit (Day 7)

A subsequent visit will be scheduled after 5-9 days of product dosing (target Day 7). Every attempt will be made to schedule appointment after 7 days of dosing to ensure adequate exposure. If a participant has discolored or malodorous vaginal discharge or suspected cervicitis at any Study Visit, an appropriate clinical and laboratory work-up will be conducted (see Appendix IV). If a participant has a genital ulcer, tests will be conducted for syphilis or herpes simplex as appropriate. Continuation in the study will be based on the severity of the abnormality noted as described in Appendix IV.

## Clinical Procedures

- Update locator information.
- Review interim medical history, AE information, and concomitant medication information.
- Review daily study records, note the time of last application of the product, and conduct adherence review.
- Count and record the number of used and unused tubes and dispose of used tubes in accordance with the guidelines.
- Perform speculum exam including
- Visual inspection of vagina and cervix,
- pH sample from vaginal wall, anterior and posterior fornices,
- Swabs for wet mount and p30 test for detection of semen,
- CVL collection.
- If abnormalities are noted, refer to Appendix IV for procedures to be followed.
- Schedule Study Visit for Day 14 and complete all required data collection forms.
- Review applicator instructions, remind participant to abstain from vaginal and anal intercourse and remind participant to continue twice daily dosing.
- Instruct participant to not apply a dose on the morning of the Day 14 Visit. The dose will be applied after the study visit to avoid the effects of residual drug on study samples.
- Instruct participant to bring used and unused products to the next visit.

## Laboratory Procedures

- Record vaginal pH measurements.
- Examine wet mount by direct microscopy to detect *T. vaginalis*, *C. albicans* infections, clue cells for BV, and/or sperm. Perform whiff test and record results.
- Conduct p30 test for detection of semen.
- Process, spin, measure volume, aliquot and freeze CVL supernatants.

## Follow-up Visit (Day 14)

A subsequent visit will be scheduled after 12-15 days of product dosing (target Day 14). Every attempt will be made to schedule appointment after 14 days of dosing to ensure adequate exposure. If a participant has discolored or malodorous vaginal discharge or suspected cervicitis at any Study Visit, an appropriate clinical and laboratory work-up will be conducted (see Appendix IV). If a participant has a genital ulcer, tests will be conducted for syphilis or herpes simplex as appropriate. Continuation in the study will be based on the severity of the abnormality noted as described in Appendix IV.

## Clinical Procedures

- Update locator information.
- Review interim medical history, AE information, and concomitant medication information.
- Review daily study records, note the time of last application of the product, and conduct adherence review.
- Count and record the number of used and unused tubes and dispose of used and/or remaining tubes in accordance with the guidelines.
- Perform speculum exam including
  - Visual inspection of vagina and cervix,
  - pH sample from vaginal wall, anterior & posterior fornices,
  - Swabs for wet mount, vaginal flora and p30 test for detection of semen,
  - CVL collection,
  - Cytobrush collection,
  - Colposcopically directed cervical biopsy.
- If abnormalities are noted, refer to Appendix IV for procedures to be followed.
- Schedule Final Visit 3-8 days later.
- Complete all required data collection forms.
- Remind participant to abstain from vaginal and anal intercourse.

## Laboratory Procedures

- Record vaginal pH measurements.
- Examine wet mount by direct microscopy to detect *T. vaginalis*, *C. albicans* infections, clue cells for BV, and/or sperm. Perform whiff test.
- Conduct p30 test for detection of semen.
- Process, spin, measure volume, aliquot and freeze CVL supernatants.
- Process cytobrush for evaluation of cell populations by FACS.
- Process cervical biopsy tissue for assessment of epithelial integrity by microscopy and qRT-PCR.
- Store vaginal swabs for vaginal flora assessment.

## Final Visit (Day 21)

## A final visit will take place 3-8 days after the final application of investigational product. This visit will mark the end of the subject’s participation in the study.

**Clinical Procedures**

- Update locator information.
- Review interim medical history, AE information, and concomitant medication information.
- Review daily study records.
- Perform speculum exam including
  - Visual inspection of vagina and cervix,
  - pH sample from vaginal wall, anterior & posterior fornices,
  - Swabs for wet mount, vaginal flora and p30 test for detection of semen,
  - CVL collection,
  - Cytobrush collection.
- If abnormalities are noted, refer to Appendix IV for procedures to be followed.
- Complete all required data collection forms.
- Complete Termination Form.

## Laboratory Procedures

- Record vaginal pH measurements.
- Examine wet mount by direct microscopy to detect *T. vaginalis*, *C. albicans* infections, clue cells for BV, and/or sperm. Perform whiff test.
- Conduct p30 test for detection of semen.
- Process, spin, measure volume, aliquot and freeze CVL supernatants.
- Process cytobrush for evaluation of cell populations by FACS.
- Store vaginal swabs for vaginal flora assessment.

## 7.2 Interim Contacts and Visits (ad hoc)

Interim contacts and visits may be performed at participant request at any time during the study. All interim contacts and visits will be documented in participants' study records and on applicable case report forms.

Some interim visits may occur for administrative reasons (for example, the participant may have questions for study staff). Interim visits may also occur in response to AEs experienced by study participants.

When interim contacts or visits are completed in response to participant reports of AEs, study clinicians will assess the reported event clinically and provide or refer the participant for appropriate medical care.

**7.3 Specimen Preparation, Handling and Shipping**

Unused drug will be shipped to Instead Healthcare, LLC and vaginal swabs for assessment of vaginal flora will be shipped to Dr. David Fredricks at the Fred Hutchinson Cancer Research Center at the University of Washington in accordance with IATA specimen shipping regulations.

**7.4 Biohazard Containment**

## Appropriate blood and secretion precautions will be employed by all personnel in the drawing of blood and shipping and handling of all specimens for this study, as currently recommended by the U.S. Centers for Disease Control and Prevention (CDC). Universal precautions will be observed at all times.

**8.0 ASSESSMENT OF SAFETY**

**8.1 Adverse Events**

The Female Genital Grading Table for Use in Microbicide Studies will be the primary tool for grading adverse events for this protocol and is available on the RCC website at http://rcc.tech-res-intl.com/eae.htm. AEs not included in that table will be graded using the Division of AIDS Table for Grading the Severity of Adult and Pediatric Adverse Events (DAIDS AE Grading Table), Version 1.0, December 2004. In cases where an AE is covered in both tables, the Female Genital Grading Table will be the grading scale utilized. Study staff will document on case report forms all AEs reported by or observed in enrolled study participants regardless of severity and presumed relationship to study product. All AE reports should contain the date the AE occurred, a brief description of the event, the time of onset, the duration of the event, the severity, the treatment required (if any), the relationship to study drug, the study drug action taken, the outcome, and whether or not the AE is Serious. The investigator or designee will assess the relationship of all AEs to the study product based on prior clinical studies, the Investigator’s Brochure, and his/her clinical judgment.

Acidform gel has 510K clearance from the FDA for use as a lubricant. This puts adverse event reporting for the study agent under device regulations. The investigator shall submit to the reviewing IRB, Safety Committee, NIAID Medical Officer, and the Center for Devices and Radiological Health (CDRH) a report of any unanticipated adverse device effect (UADE) occurring during the study as soon as possible, but in no event later than 10 working days after the investigator first learns of the effect. Unanticipated adverse device effect means any serious adverse effect on health or safety or any life-threatening problem or death caused by, or associated with, a device, if that effect, problem, or death was not previously identified in nature, severity, or degree of incidence in the investigational plan or application (including a supplementary plan or application), or any other unanticipated serious problem associated with a device that relates to the rights, safety, or welfare of subjects. For reporting to CDRH, the contact information is:

Sheila Brown

POS/IDE, HFZ-401

FDA, CDRH

9200 Corporate Blvd.

Rockville, MD 20850

**8.2 Local regulatory requirements**

Information on all AEs experienced by study participants will be included in reports to the DAIDS Medical Officer. Study staff will report information on AEs to the AECOM IRB and GCRC in accordance with all applicable regulations and site-specific requirements.

As required by New York State law, all positive results for chlamydia, gonorrhea, HIV and syphilis will be reported to the New York City and State Departments of Health.

**8.3 Social harms reporting**

Participants may become embarrassed, worried, or anxious when completing their HIV risk assessment and/or receiving HIV counseling. They also may become worried or anxious while waiting for their HIV test results. Trained counselors will be available to help participants deal with these feelings.

Although the study site will make every effort to protect participant privacy and confidentiality, it is possible that participants' involvement in the study could become known to others, and that social harms may result (i.e., because participants could become labeled as HIV-infected or at "high risk" for HIV infection). For example, participants could be treated unfairly or discriminated against, or could have problems being accepted by their families and/or communities.

Women in abusive relationships may be unable to avoid sexual activity due to risk of physical harm. The study team will carefully inquire whether participants are able to decline intercourse and will not enroll any subjects who are evasive or uncertain in their responses.

## 9.0 CLINICAL MANAGEMENT

**9.1 Toxicity Management**

In response to AEs reported by study participants and/or observed upon exam by study staff, the study site investigator or designee will recommend either continuation or holding gel use consistent with the criteria in Appendix IV.

**9.2 Other Disease Events**

Study staff will comply with all applicable local requirements to report communicable diseases including HIV and other reportable STI identified among study participants and their partners (if obligated by local law) to local health authorities. Participants will be made aware of all reporting requirements during the study informed consent process. As required by New York State law, all positive results for chlamydia, gonorrhea, HIV and syphilis will be reported to the New York City and State Departments of Health.

**9.3 Pregnancy**

If a participant is found to be pregnant at the screening or enrollment visits, she will discuss the results with the clinician and will not continue in the study. She will be referred for care by the study clinicians. If she becomes pregnant during the course of the study, she will discontinue study product and be referred for care. The study team will attempt to obtain pregnancy outcome data on any women who become pregnant while on study drug.

## 9.4 Breastfeeding

Currently, it is unknown if there are any effects of Acidform lubricant on breast-milk. Though it is unlikely that the gel will pass through breast milk, systemic absorption of the gel into the blood may affect breast milk and cause harm to an infant. Therefore, participants who are breastfeeding will be excluded from participation in the study.

**9.5 Criteria for Discontinuation**

Accrual into this study will be suspended if two or more participants in the same arm experience similar grade 3 or higher AEs (as defined by the Female Genital Grading Table for Use in Microbicide Studies and the DAIDS Table for Grading the Severity of Adult and Pediatric Adverse Events, Version 1.0, December 2004) judged possibly, probably, or definitely related to product use. The protocol team in collaboration with the Safety Committee and NIH NIAID/DAIDS Medical Officer then will review all pertinent data and determine whether to continue accrual and product use. The team may decide to stop the study at this time, or at any such time that the team agrees that an unacceptable type and/or frequency of AEs have been observed.

The study may be discontinued at any time by the IRB, the NIH, the pharmaceutical supporters or designees, the Office for Human Research Protections (OHRP), or other government agencies as part of their duties to ensure that research subjects are protected.

Monitoring of AEs will be ongoing through regular reviews (safety monitoring). If needed, the protocol team will convene ad hoc meetings in the event of an abnormal number of reported AEs judged definitely, probably, possibly, or probably not related to study gel or applicator, or any other conditions deemed as an emergency event by team members.

**9.6 Criteria for Permanent Treatment Discontinuation for an Individual Participant**

The criteria for permanent treatment discontinuation of further study product/intervention(s) for an individual participant are:

- Treatment-related toxicity (see section 9), including pelvic inflammatory disease, deep epithelial disruption
- Requirement for prohibited concomitant medications such as oral or intravenous antibiotics
- Pregnancy or breastfeeding
- Completion of treatment as defined in the protocol
- Request by participant to terminate treatment
- Clinical reasons believed life-threatening by the physician even if not addressed in the toxicity section of the protocol
- Unwillingness or inability to remain abstinent for the duration of the study.

The participant will continue to be followed with participant’s permission if study product/intervention(s) is discontinued.

**9.7 Criteria for Premature Study Discontinuation for an Individual Participant**

The criteria for premature discontinuation from the study for an individual participant are:

- Pregnancy or breastfeeding
- Request by participant to withdraw
- Request of the primary care provider if s/he thinks the study is no longer in the best interest of the participant
- Participant judged by the investigator to be at significant risk of failing to comply with the provisions of the protocol as to cause harm to self or seriously interfere with the validity of study results
- At the discretion of the IRB, OHRP, NIH, investigator, or pharmaceutical supporter.

## 10.0 STATISTICAL CONSIDERATIONS

## 10.1 Overview and Statistical Designs Issues

## All subjects who receive treatment (Acidform lubricant or HEC placebo gel) will be included in all analyses. The primary objective of this study is to compare the safety of Acidform lubricant to HEC gel by assessing the genital tract mucosal response to 14 days of twice daily application by measuring endogenous antimicrobial activity and levels of mediators of host defense, including defensins, cytokines and chemokines in CVL. The clinical safety of Acidform when administered twice daily for 14 consecutive days will be assessed by also including the following endpoint: Grade 2 or higher adverse experiences judged by the Investigator to be possibly, probably, or definitely related to product use.

Differences between groups with respect to age and baseline amounts of immune mediators will be compared by *t* test. Differences between groups with respect to endogenous antimicrobial activity of CVL against HSV and *E. coli* and change in mucosal immune mediators will be compared by analysis of covariance and adjusted for baseline (after log transformation of data for cytokines, chemokines, and mucosal mediators). Differences between groups with respect to change in percentage reduction in viral or bacterial infection after application of drug or placebo gel relative to titers in screening CVL samples will be determined by Wilcoxon nonparametric test (with exact p-values).

All visits in which a woman has been exposed to the study product will be included in the analysis of clinical safety. The number and the percentages of participants experiencing at least one Grade 2 or higher AE, and the number and percentage experiencing each specific AE will be tabulated by study arm. Each participant will contribute once in each category (i.e. only for highest severity AE for each participant) for the calculation of event rates. The number and percentage of participants experiencing a Grade 2 or higher AE will be tabulated by severity and relationship to treatment for each treatment group. AEs that lead to study product discontinuation will be listed in a separate data listing. The number and percentage of participants with a Grade 2 or higher AE judged possibly, probably or definitely related to study treatment will be summarized and compared for each treatment group by Fisher’s exact test.

An additional objective is to assess whether a single dose of Acidform lubricant or HEC present in CVL 2 hours after intravaginal application reduces HIV and HSV *in vitro*. Differences between the groups with respect to log reduction in HIV or HSV infection after application of Acidform or HEC relative to titers in baseline CVL samples will be determined by Wilcoxon nonparametric test (with exact p values).

Dr. Hillel W. Cohen will provide statistical support through the Biostatistical Core at the GCRC and the Institute for Clinical and Translational Research at AECOM/MMC. Statistical analyses will be performed with STATA software (Version 10, STATA Corp.).

**10.2 Sample Size Considerations**

There is limited data regarding intra- and inter-subject variability of endogenous antimicrobial activity detectable in CVL. We recently completed a pilot study that included measuring endogenous antimicrobial activity and immune mediators in CVL collected weekly for 2 months from healthy women who were either naturally cycling or receiving hormonal contraception. Among 9 cycling women, the mean percentage inhibition of HSV infection (defined as the viral plaque formation in cells treated with CVL as a percentage of plaque formation in the presence of control fluid) at the initial visit was 54 with a standard deviation (sd) of 28. The sd is the same if we use the mean of all samples collected from the cyclers (n=52). If we assume that Acidform and HEC have no impact on endogenous anti-HSV activity and consider 28 (1 sd) as functionally equivalent, a sample size of 18 subjects per arm will provide 80% power to observe statistically significant equivalence of means. This sample size is similar to other Phase I studies of topical microbicide products.

The following table represents the probability of observing zero, at least one, and two or more adverse experiences among the sample size of 18 using Acidform for various “true” event rates:

**Analysis of Adverse Event Frequency with n = 18**

| **Event Rate** | **P (0 events | n=18)** | **P (>1 event | n=18)** | **P (>2 events | n=18)** |
| --- | --- | --- | --- |
| 1% | 0.83 | 0.17 | 0.01 |
| 5% | 0.40 | 0.60 | 0.23 |
| 10% | 0.15 | 0.85 | 0.55 |
| 15% | 0.05 | 0.95 | 0.78 |
| 25% | <0.01 | >0.99 | 0.96 |
| 35% | <0.01 | >0.99 | >0.99 |
| 45% | <0.01 | >0.99 | >0.99 |

For example, if the true rate of an adverse experience is five percent, the probability that the endpoint will be observed in at least one of the 18 women exposed to Acidform is 0.60.

**10.3 Enrollment/Randomization/Blinding Procedures**

The randomization will be 1:1 (i.e., 18 in each treatment arm). The AECOM Research Pharmacy will provide the randomization procedure for both clinical sites. Subjects will be given the choice of selecting a clinical site. A specific number of women is not required at each site. Throughout the period of study implementation and data analysis, the Herold laboratory team will not be informed of the participants’ random assignments. Although the clinical study team will be aware of the participants’ assignment to either Acidform or HEC, the lab staff will perform all proposed testing blindly.

Both study gels will be supplied in single-use tubes, and single-use applicators packaged in individual wrappers. Lab staff will be unblinded after all study visits and data analyses are completed.

**10.4 Participation Enrollment and Follow-Up**

Thirty-six healthy women will be enrolled in this study. Participant accrual will last approximately eighteen months in this study and each subject’s participation in this study will last approximately two months. Once a participant has enrolled in the study, the study site will make every reasonable effort to retain her for the complete study period. Given the relatively small study sample size, and the importance of ascertaining all outcomes among study participants, 100 percent retention of enrolled participants is targeted. If a subject drops out after any visit, she will be replaced.

## 10.5 Data and Safety Monitoring

Close collaboration among protocol team members will be necessary to evaluate study progress and respond to occurrences of toxicity in a timely manner. Rates of accrual, adherence, follow-up, and AE incidence will be monitored closely by the protocol team on a regular basis. The team will meet weekly during the period of study implementation.

A Safety Committee composed of individuals unaffiliated with the study has been formed. The committee was carefully selected based on scientific and clinical backgrounds and has expertise in virology and clinical trials. Two physicians have been identified who will serve in this function well. Kathy Anastos, M.D. is the Principal Investigator (PI) of the multi-center Women’s Interagency HIV Study (WIHS). Karen Beckerman, M.D. has extensive experience in HIV and gynecology. The Committee will review safety and trial progress and provide advice with respect to study continuation, modification and/or termination. In order to achieve a timely evaluation of participant safety, the PI will submit a brief report to the Safety Committee every 3 months during the study. All serious adverse events will be reported to the Committee, the AECOM IRB, Instead Healthcare, LLC and the NIH NIAID/DAIDS Medical Officer within 24 hours of diagnosis.  The Safety Committee will review these data for safety parameters at least every 3 months or more often as necessary (for instance if any serious adverse event occurs).  The Committee will organize a meeting if toxicity becomes an issue in between scheduled semi-annual meetings.  The Committee will report any safety concerns to the PI, AECOM IRB and the NIH NIAID/DAIDS Medical Officer.

**10.6 Safety Review**

The PI is responsible for continuous close monitoring of all AEs that occur among study participants. For this study, accrual will be suspended if two or more study participants in the same arm experience similar grade 3 or higher AEs (as defined by the Female Genital Grading Table for Use in Microbicide Studies and the DAIDS Table for Grading Severity of Adverse Experiences) judged possibly, probably, or definitely related to product use. The protocol team, in collaboration with the Safety Committee and NIH NIAID/DAIDS Medical Officer, will review all pertinent data and determine whether to continue accrual and product use. A decision to stop the study may be made by the team at this time, or at any such time that the team agrees that an unacceptable type and/or frequency of AEs has been observed.

## 11.0 DATA HANDLING AND RECORDKEEPING

**11.1 Data Management Responsibilities**

The study will be conducted in full compliance with the protocol. The protocol team will develop study case report forms. Data management will be performed at AECOM. Close collaboration among protocol team members will be necessary to evaluate study progress and respond to occurrences of toxicity in a timely manner. Rates of accrual, adherence, follow-up, and AE incidence will be monitored closely by the protocol team on a regular basis. The team will meet weekly during the period of study implementation. In addition, close cooperation between the protocol team members, personnel from NIH and pharmaceutical supporters will be necessary in order to track study progress, respond to queries about proper study implementation, and address other issues in a timely manner. Assignment of all sponsor responsibilities for this study will be specified in a Clinical Trials Agreement executed between Instead Healthcare, LLC and the Albert Einstein College of Medicine.

**11.2 Source Documents and Access to Source Data/Documents**

The Investigator will maintain, and store in a secure manner, complete, accurate, and current study records throughout the study. Study records include administrative documentation — including site registration documents and all reports and correspondence relating to the study — as well as documentation related to each participant screened and/or enrolled in the study — including informed consent forms, locator forms, case report forms, notations of all contacts with the participant, and all other source documents. All records must be retained on-site throughout the study’s period of performance. The investigator will allow inspection of all study-related documentation by authorized representatives of NIAID, Instead Healthcare, LLC and regulatory agencies to examine (and when required by applicable law, to copy) clinical records for the purposes of quality assurance reviews, audits, and evaluation of the study safety and progress. Study records will not be destroyed without approval from the NIH.

## 12.0 HUMAN SUBJECTS PROTECTION

## 12.1 Institutional Review Board

## The protocol, informed consent forms, and recruitment materials, and other requested documents — and any subsequent modifications — will be reviewed and approved by the IRB at the AECOM. As of October 23, 2007, in accordance with each institution’s Federalwide Assurances, the Albert Einstein College of Medicine of Yeshiva University (FWA 00000140) and the Montefiore Medical Center (FWA 00002558) entered into an agreement that permits the IRB of either institution to be designated as the sole IRB for research taking place at both institutions. IRB transactions are reviewed and approved by the designated IRB, which for this study is the AECOM IRB. Following IRB review and approval, the study sites will submit required documents to complete protocol registration in accordance with DAIDS procedures. Subsequent to initial review and approval, the IRB will review the protocol at least annually. The PI will make safety and progress reports to the IRB and NIH at least annually, and within three months of study termination or completion. These reports will include the total number of participants enrolled in the study, the number of participants who completed the study, all changes in the research activity, and all unanticipated problems involving risks to human subjects or others.

## 12.2 Informed Consent Process

Written informed consent will be obtained from each study participant before she is screened for the study. All participants also must verbally affirm their understanding of the investigational nature of the study, its risks and benefits, other treatment alternatives, their rights to terminate participation in the study without affecting their health care at the study site, whom to contact with questions regarding the study, and that he or she freely has given informed consent to participate in the study. A separate consent form will be obtained from each participant for HIV testing. Copies of the informed consent and HIV consent will be given to each participant and this fact will be documented in the participant’s record.

## 12.3 Participant Confidentiality

All study-related information will be stored securely at the study site. All participant information will be stored in locked file cabinets in areas with access limited to study staff. All laboratory specimens, reports, study data collection, process, and administrative forms will be identified by coded number only to maintain participant confidentiality. All computer entry and networking programs also will be done by coded number only, and all local databases will be secured with password-protected access systems. Forms, lists, logbooks, appointment books, and any other listings that link participant ID numbers to other identifying information will be stored in a separate, locked file in an area with limited access.

## Documentation, data and all other information generated will be held in strict confidence. No information concerning the study or the data will be released to any unauthorized third party, without prior written approval of the participant except as necessary for monitoring by the IRB, the study sponsor, the OHRP, and the pharmaceutical supporters.

##

**12.4 Study Discontinuation**

Once a participant has enrolled in the study, the study site will make every reasonable effort to retain her for the complete study period. Given the relatively small study sample size, and the importance of ascertaining all outcomes among study participants, 100 percent retention of enrolled participants is targeted.

However, participants may withdraw from the study for any reason at any time. The Principal Investigator (PI) also may withdraw participants from the study in order to protect their safety and/or if they are unwilling or unable to comply with required study procedures.

During the study, participants with any abnormal condition will be treated according to standard clinical practice. A participant with a clinical toxicity outcome (see Appendix IV) who is discontinued from further product use will be considered to have reached a study endpoint. Participants also may be withdrawn if the study sponsor or regulatory authorities terminate the study prior to its planned end date.

Every reasonable effort will be made to complete a final evaluation of participants who terminate from the study prior to completion, and study staff will record the reason(s) for all withdrawals from the study in participants’ study records.

If at the screening or enrollment visits, a participant is found to have an abnormality on pelvic exam or a urinary tract infection, she will be ineligible to enroll in the study. Following treatment for candida vaginitis, bacterial vaginosis or urinary tract infection, she may be eligible to be re-screened for participation in the study. A minimum interval of one month is required between treatment and re-screening and all exclusion criteria will apply. If a participant is found to be pregnant, she will be referred for care.

Accrual into this study will be suspended if two or more participants in the same arm experience similar grade 3 or higher AEs (as defined by the Female Genital Grading Table for Use in Microbicide Studies and the DAIDS Table for Grading Severity of Adverse Experiences) judged possibly, probably, or definitely related to product use. The protocol team in collaboration with the Safety Committee and NIH Medical Officer then will review all pertinent data and determine whether to continue accrual and product use. The team may decide to stop the study at this time, or at any such time that the team agrees that an unacceptable type and/or frequency of AEs have been observed.

The study also may be discontinued at any time by NIH, Instead Healthcare, LLC and/or the OHRP or other regulatory authorities.

## 13.0 PUBLICATION POLICY

## Publication of the results of this study will be governed by DAIDS policies. Any presentation, abstract, or manuscript will be submitted by the Investigator to DAIDS for review prior to submission.

## 14.0 REFERENCES

## 1. 2007 AIDS Epidemic Update. In: Organization JUNPoHAaWH, ed; 2007.

## 2. Update: barrier protection against HIV infection and other sexually transmitted diseases. *MMWR*. Vol. 42; 1993:589-597.

## 3. Elias CJ, Coggins C. Female-controlled methods to prevent sexual transmission of HIV. *AIDS*. 1996;10 Suppl 3:S43-51.

## 4. Van Damme L, Ramjee G, Alary M, et al. Effectiveness of COL-1492, a nonoxynol-9 vaginal gel, on HIV-1 transmission in female sex workers: a randomised controlled trial. *Lancet*. 2002;360(9338):971-7.

## 5. Doncel G, Van Damme L. Update on the CONRAD cellulose sulfate trial. 14th Conference on Retroviruses and Opportunistic Infections. Los Angeles, CA; 2007.

## 6. Ramjee G, Govinden R, Morar NS, Mbewu A. South Africa's experience of the closure of the cellulose sulphate microbicide trial. *PLoS Med*. 2007;4(7):e235.

## 7. Investigator's Brochure: Acidform Gel. 2004.

## 8. Garg S, Anderson RA, Chany CJ, 2nd, et al. Properties of a new acid-buffering bioadhesive vaginal formulation (ACIDFORM). *Contraception*. 2001;64(1):67-75.

## 9. Connor RI. Sensitivity of non-clade B primary HIV-1 isolates to mildly acidic pH. *J Acquir Immune Defic Syndr*. 2006;43(4):499-501.

## 10. Tuyama AC, Cheshenko N, Carlucci MJ, et al. ACIDFORM inactivates herpes simplex virus and prevents genital herpes in a mouse model: optimal candidate for microbicide combinations. *J Infect Dis*. 2006;194(6):795-803.

## 11. Keller MJ, Guzman E, Hazrati E, et al. PRO 2000 elicits a decline in genital tract immune mediators without compromising intrinsic antimicrobial activity. *AIDS*. 2007;21(4):467-76.

## 12. Keller MJ, Zerhouni-Layachi B, Cheshenko N, et al. PRO 2000 gel inhibits HIV and herpes simplex virus infection following vaginal application: a double-blind placebo-controlled trial. *J Infect Dis*. 2006;193(1):27-35.

## 13. Patel S, Hazrati E, Cheshenko N, et al. Seminal plasma reduces the effectiveness of topical polyanionic microbicides. *J Infect Dis*. 2007;196(9):1394-402.

## 14. Fuchs EJ, Lee LA, Torbenson MS, et al. Hyperosmolar sexual lubricant causes epithelial damage in the distal colon: potential implication for HIV transmission. *J Infect Dis*. 2007;195(5):703-10.

## 15. Schwartz JL, Mauck C, Lai JJ, et al. Fourteen-day safety and acceptability study of 6% cellulose sulfate gel: a randomized double-blind Phase I safety study. *Contraception*. 2006;74(2):133-40.

## 16. Fredricks DN, Fiedler TL, Marrazzo JM. Molecular identification of bacteria associated with bacterial vaginosis. *N Engl J Med*. 2005;353(18):1899-911.

## 17. Fredricks DN, Fiedler TL, Thomas KK, Oakley BB, Marrazzo JM. Targeted PCR for detection of vaginal bacteria associated with bacterial vaginosis. *J Clin Microbiol*. 2007;45(10):3270-6.

## 18. Amaral E, Perdigao A, Souza MH, et al. Postcoital testing after the use of a bio-adhesive acid buffering gel (ACIDFORM) and a 2% nonoxynol-9 product. *Contraception*. 2004;70(6):492-7.

## 19. Schwartz JL, Poindexter A, Schmitz SW, Mauck C, Callahan MM. Male tolerance of ACIDFORM gel. *Contraception*. 2005;71(6):443-6.

## 20. Tien D, Schnaare RL, Kang F, et al. In vitro and in vivo characterization of a potential universal placebo designed for use in vaginal microbicide clinical trials. *AIDS Res Hum Retroviruses*. 2005;21(10):845-53.

## 21. Schwartz JL, Ballagh SA, Kwok C, et al. Fourteen-day safety and acceptability study of the universal placebo gel. *Contraception*. 2007;75(2):136-41.

**Appendix I. Schedule of assessments**

|  | **Screening** | **Enrollment**  **(Day 0)** | **Day 7**  **(Day 5-9)** | **Day 14**  **(Day 12-15)** | **Final Visit (Day 21)**  **(Day 16-21)** |
| --- | --- | --- | --- | --- | --- |
| Informed Consent | X |  |  |  |  |
| Sexual History | X | X*^b^* | X*^b^* | X*^b^* | X*^b^* |
| Medical History/Concomitant Meds | X | X*^b^* | X*^b^* | X*^b^* | X*^b^* |
| HIV EIA with confirmation | X |  |  |  |  |
| Provide HIV/STI pre-test counseling | X |  |  |  |  |
| Provide HIV/STI test results and post-test counseling |  | X |  |  |  |
| Urine Microscopy/Culture | X |  |  |  |  |
| Urine pregnancy test | X | X |  |  |  |
| Serum pregnancy test | X |  |  |  |  |
| Pelvic Exam | X | X | X | X | X |
| Pap test*^c^* | X |  |  |  |  |
| Vaginal pH | X | X | X | X | X |
| Wet Mount (*Trichomonas*, *Candida*, BV) | X | X | X | X | X |
| Herpes simplex virus culture | X*^a^* | X*^a^* | X*^a^* | X*^a^* | X*^a^* |
| ABAcard p30 test for detection of semen | X | X | X | X | X |
| *N. gonorrhoeae/C. trachomatis* | X | X*^a^* | X*^a^* | X*^a^* | X*^a^* |
| *T. pallidum* (RPR) with confirmation | X |  |  |  |  |
| Herpes simplex virus serology | X |  |  |  |  |
| CVL collection | X | X | X | X | X |
| Cytobrush collection |  | X |  | X | X |
| Vaginal swab to assess vaginal flora |  | X |  | X | X |
| Cervical biopsy | X |  |  | X |  |
| Record Adverse Events |  | X | X | X | X |
| Distribute study supplies |  | X |  |  |  |
| Distribute Participant Diary |  | X |  |  |  |
| Review Participant Diary |  |  | X | X | X |
| Count used & unused supplies |  |  | X | X |  |

*^a^*If clinically indicated

*^b^*Interim history collected

^c^A Pap test will be obtained if documentation of a negative result within the last year is not available

**Appendix II. Laboratory Evaluations**

| **Test** | **Purpose** | **Specimen** | **Volume** | **Laboratory** |
| --- | --- | --- | --- | --- |
| HCG | Pregnancy | Urine | 10 ml | GCRC |
| Urine microscopy and culture | Urinary tract pathogens | Urine | 10 ml | MMC/AECOM |
| Wet mount | *Trichomonas*, *Candida*, BV, sperm | Swab/slide | n/a | GCRC |
| pH | Vaginal pH | Stainless steel probe | n/a | GCRC |
| ABAcard p30 | Semen detection | Swab | n/a | GCRC |
| GC/CT | *N. gonorrhoeae/ C. trachomatis* | Swab | 1 | MMC/AECOM |
| EIA with confirmation | HIV | Red Top | 5 ml | MMC/AECOM |
| RPR with confirmation | T. pallidum | Red Top | 3 ml | MMC/AECOM |
| Serum HCG | Pregnancy test | Red Top | 2 ml | MMC/AECOM |
| Pap test | Cancer screening | Liquid preservative | 1 | MMC/AECOM |
| FACS | Immune cell populations | Cytobrush | n/a | Investigator Lab |
| Multiplex proteome array | Cytokines, chemokines | CVL supernatant | 3-5 ml | Investigator Lab |
| ELISA | SLPI, defensins, lactoferrin, lysozyme, IL-1ra, IgG, IgA | CVL supernatant | 1 ml | Investigator Lab |
| PCR | Vaginal flora | Swab | n/a | Investigator Lab |
| Cervical biopsy | Epithelial integrity | Tissue | n/a | Investigator Lab |

##

## Appendix III: Instructions for Using Acidform Lubricant or HEC gel

The study kit provided to you contains tubes of the study gel and applicators to insert the gel into the vagina. Before using the gel and applicators, please read the following instructions carefully. The study nurse will answer any questions you may have.

1. Remove one applicator and one tube of study gel from the study kit.

2. Remove applicator from the sealed wrapper. Before filling the applicator, push plunger completely down inside barrel.

3. To open the tube, unscrew the plastic cap. Turn the cap upside down and puncture the foil seal on the opening of the tube.

4. Holding the tube upright, attach the applicator barrel to the neck of the tube and turn the applicator clockwise until it is firmly seated.

5. Hold the applicator firmly on the tube neck. Squeeze the tube from the bottom filling the applicator to the single line indicated.

6. Unscrew the study gel tube from the applicator.

7. With one hand, hold the filled applicator by placing your thumb and middle finger HALF-WAY along the barrel.

8. Choose a comfortable position, for example, standing with one leg raised, squatting with your feet apart, or lying on your back with your knees apart. With the other hand, gently spread the vaginal opening.

9. GENTLY slide the applicator into the vagina until your fingers touch your body. Be careful not to insert the applicator too far.

10. Hold the applicator firmly in place and push the plunger into the barrel (the gel-filled half of the applicator). With the plunger still depressed, withdraw the applicator from the vagina.

11. Replace plastic cap on study gel tube and store the used tube in the study kit. Bring the study kit with you on your next visit. Store in an area not accessible to children. Product should be stored at controlled room temperature until required for administration. Controlled room temperature is defined as 25 degrees Celsius (°C)/ 77 degrees Fahrenheit (°F).

12. Used applicators should be stored in the study kit and should be brought with you to your next visit.

## Appendix IV: Outcomes, Diagnostics, and Follow-Up Procedures

Individual participants may withdraw from study participation at any time, for any reason, without loss of other benefits or services to which they may be entitled. Participants with any abnormal condition will be treated according to standard clinical practice.

| **Condition** | **Product Use** | **Evaluation** | | **Enrollment Action** | | **Follow-up and Treatment Action** | |
| --- | --- | --- | --- | --- | --- | --- | --- |
| Ulceration | Adverse clinical event, discontinue product use | Culture for genital herpes. |  | | Re-evaluate in 5 to 7 days. If the ulcer has become worse or not healed in 5 to 7 days, perform a biopsy. Ask the participant to return in 7 to 10 days for follow-up. | |  |
| Abrasion | Continue |  |  | | Re-evaluate by speculum examination in 48 to 72 hours. If the condition is worse, discontinue product use. If the condition is the same, continue product use. | |  |
| Generalized erythema or severe edema: A localized area of more than 30% of vulvar surface or combined vaginal and cervical surface affected by erythema | Adverse clinical event, discontinue product use |  |  | | Re-evaluate in 5 to 7 days. | |  |
| Vaginitis | Adverse clinical event, discontinue product use | Perform wet mount for *candida vaginitis, trichomoniasis,* and bacterial vaginosis. |  | | Manage with oral medication and re-evaluate in seven days. If the participant has asymptomatic candida vaginitis at the Day 7 visit she should continue product use and be re-evaluated at the Day 14 visit.[[1]](#footnote-2) | |  |

| Suspected cervicitis | Provisionally continue | Evaluate for *N. gonorrhoeae and C. trachomatis*. Perform wet mount for  *trichomoniasis.* | If laboratory results confirm pathogen, discontinue treatment. | Treat with oral medication and re-evaluate in 5 to 7 days. |
| --- | --- | --- | --- | --- |
| Petechial hemorrhage | Continue |  |  | Re-evaluate by speculum examination in 48 to 72 hours. If the condition is worse, discontinue product use. If the condition is the same, continue product use. |
| Ecchymosis | Continue |  |  | Re-evaluate by speculum examination in 48 to 72 hours. If the condition is worse, discontinue product use. If the condition is the same, continue product use. |
| Sub-epithelial hemorrhage with swelling | Continue |  |  | Re-evaluate by speculum examination in 48 to 72 hours. If the condition is worse, discontinue product use. If the condition is the same, continue product use. |
| Mild to moderate erythema or edema: A localized area of less than 30% of vulvar surface or combined vaginal and cervical surface | Continue |  |  | Re-evaluate by speculum examination in 48 to 72 hours. If the condition is worse, discontinue product use. If the condition remains the same, continue product use. |
|  |  |  |  |  |

1. If at the Day 21 visit there are signs and symptoms compatible with vaginitis, treat and follow-up to document resolution. If the wet mount indicates *candida vaginitis*, but the participant is still asymptomatic, schedule a follow-up visit one to two weeks post-study. Record wet mount findings and clinical status at that time. [↑](#footnote-ref-2)
